# Photoacoustic Tomography of Human Hepatic Malignancies Using Intraoperative Indocyanine Green Fluorescence Imaging

**DOI:** 10.1371/journal.pone.0112667

**Published:** 2014-11-07

**Authors:** Akinori Miyata, Takeaki Ishizawa, Mako Kamiya, Atsushi Shimizu, Junichi Kaneko, Hideaki Ijichi, Junji Shibahara, Masashi Fukayama, Yutaka Midorikawa, Yasuteru Urano, Norihiro Kokudo

**Affiliations:** 1 Hepato-Biliary-Pancreatic Surgery Division, Department of Surgery, Graduate School of Medicine, the University of Tokyo, Tokyo, Japan; 2 Laboratory of Chemical Biology and Molecular Imaging, Graduate School of Medicine, the University of Tokyo, Tokyo, Japan; 3 Department of Gastroenterology, Graduate School of Medicine, the University of Tokyo, Tokyo, Japan; 4 Department of Pathology, Graduate School of Medicine, the University of Tokyo, Tokyo, Japan; 5 Genome Science Division, Research Center for Advanced Science & Technology, the University of Tokyo, Tokyo, Japan; National Cancer Institute, United States of America

## Abstract

Recently, fluorescence imaging following the preoperative intravenous injection of indocyanine green has been used in clinical settings to identify hepatic malignancies during surgery. The aim of this study was to evaluate the ability of photoacoustic tomography using indocyanine green as a contrast agent to produce representative fluorescence images of hepatic tumors by visualizing the spatial distribution of indocyanine green on ultrasonographic images. Indocyanine green (0.5 mg/kg, intravenous) was preoperatively administered to 9 patients undergoing hepatectomy. Intraoperatively, photoacoustic tomography was performed on the surface of the resected hepatic specimens (n = 10) under excitation with an 800 nm pulse laser. In 4 hepatocellular carcinoma nodules, photoacoustic imaging identified indocyanine green accumulation in the cancerous tissue. In contrast, in one hepatocellular carcinoma nodule and five adenocarcinoma foci (one intrahepatic cholangiocarcinoma and 4 colorectal liver metastases), photoacoustic imaging delineated indocyanine green accumulation not in the cancerous tissue but rather in the peri-cancerous hepatic parenchyma. Although photoacoustic tomography enabled to visualize spatial distribution of ICG on ultrasonographic images, which was consistent with fluorescence images on cut surfaces of the resected specimens, photoacoustic signals of ICG-containing tissues decreased approximately by 40% even at 4 mm depth from liver surfaces. Photoacoustic tomography using indocyanine green also failed to identify any hepatocellular carcinoma nodules from the body surface of model mice with non-alcoholic steatohepatitis. In conclusion, photoacoustic tomography has a potential to enhance cancer detectability and differential diagnosis by ultrasonographic examinations and intraoperative fluorescence imaging through visualization of stasis of bile-excreting imaging agents in and/or around hepatic tumors. However, further technical advances are needed to improve the visibility of photoacoustic signals emitted from deeply-located lesions.

## Introduction

In vivo fluorescence imaging using indocyanine green (ICG) has been clinically applied as an intraoperative navigation tool that enables the real-time identification of biological structures, such as the lymphatic system [1], [2] and the biliary ducts [3]–[7], as well as the evaluation of visceral blood perfusion [8]–[11]. Since 2008, ICG fluorescence imaging has also been used to identify hepatic tumors during open hepatectomy [12]–[15] and, more recently, laparoscopic hepatectomy [16]. This technique is based on the accumulation of ICG, which is intravenously administered for preoperative liver function testing, in hepatocellular carcinoma (HCC) tissue and in the non-cancerous hepatic parenchyma located around adenocarcinoma foci, such as colorectal liver metastases (CRLM) and intrahepatic cholangiocarcinoma (ICC) [12]. When fluorescence images of the cut surfaces of resected specimens are obtained, well- to moderately-differentiated HCC shows uniform fluorescence of ICG in the cancerous tissue, while poorly differentiated HCC and adenocarcinoma show rim-type fluorescence around the tumor [17]. Although both types of cancer-associated ICG fluorescence can be detected by commercially-available fluorescence imaging systems as far as the tumors are located beneath the liver surface, there is a need for novel imaging technology that enables the detection of deeply-located hepatic tumors and the visualization of ICG accumulation in and/or around these lesions on cross-sectional images.

In the last decade, photoacoustic (PA) tomography has been actively developed as a novel optical imaging technology that enables the real-time visualization of deeply-located biologic structures on ultrasonographic images through the “photoacoustic effect” [18]–[20]. With this technique, nanosecond laser pulses are transmitted into tissue and absorbed by endogenous chromophores or exogenous molecular imaging agents in targeted structures. The rapid absorption increases focal temperature and produces a thermoelastic expansion that creates acoustic waves. These photoacoustic signals can be detected using ultrasound (US) receivers and used to reconstruct images of the targeted biological structures according to the absorbed optical energy density. Because ICG has a moderate fluorescence quantum yield, it can be used as a contrast agent in PA imaging in addition to fluorescence imaging [21]–[23], and its spatial distribution in cancerous and non-cancerous hepatic tissues may be visualized with both modalities. The aim of this study was to evaluate the ability of PA tomography using ICG as a contrast agent to produce representative fluorescence images of hepatic tumors, using both surgically-resected human hepatic samples and a mouse model of HCC (mice with non-alcoholic steatohepatitis [NASH]).

## Materials and Methods

This study was conducted with the approval of the Institutional Ethics Review Board of the University of Tokyo Hospital. Written informed consent was obtained from all patients. The animal study was carried out in strict accordance with the recommendations in the Guide for the Care and Use of Laboratory Animals from the National Institutes of Health. The protocol for the animal study was approved by the University of Tokyo's Committee on the Ethics of Animal Experiments. All surgery was performed under isoflurane anesthesia, and all possible efforts were made to minimize suffering.

### Establishment of PA tomography's ability to visualize ICG-containing tissue

First, the ability of PA tomography to detect ICG-containing biological material was evaluated using a human liver tissue-mimicking phantom (OST Co., Ltd., Chiba, Japan [24]) with spherical air holes 5 mm in diameter located 5 mm deep to the surface; the model was created with a three-dimensional printer (FASOTEC Co., Ltd., Chiba, Japan). Each hole was filled with human plasma containing ICG at concentrations of 0.001, 0.01, 0.1, and 1.0 mg/mL, and PA images were obtained with the Vevo LAZR imaging system (VisualSonics, Toronto, ON, Canada) as described elsewhere [25], [26]. For each ICG solution, the PA signal amplitude was measured and compared with the solution's fluorescence intensity as measured with a Maestro imaging system (CRI, Woburn, Massachusetts, USA) using the near-infrared filter setting (excitation, 730–740 nm; emission, 810 nm long pass) [17].

Next, PA tomography's ability to visualize ICG-containing cancerous tissue was confirmed using a previously-established mouse model with subcutaneously implanted human HuH-7 well-differentiated hepatoma cells (Japanese Collection of Research Bioresources Cell Bank, Osaka, Japan) [27], [28]. Photoacoustic imaging of the subcutaneous tumors was performed from the skin surface 48 hours after the intravenous injection of ICG (5 mg/kg) via the tail vein.

### Photoacoustic tomography of surgically-resected hepatic tissue in humans

In 9 patients who underwent hepatic resection for malignancies (10 nodules: HCC, n = 5; CRLM, n = 4; and ICC, n = 1), ICG (Diagnogreen, Daiichi Sankyo, Tokyo, Japan) was preoperatively injected (dose, 0.5 mg per kg of body weight; intravenous injection) as part of routine liver function testing performed for surgical planning [12], [17]. Intraoperatively, the ICG retained in hepatic tissues was utilized as a contrast agent in PA tomography as well as for fluorescence imaging. Following hepatectomy, PA images of the resected specimens were obtained, and PA signal amplitude was measured with the Vevo LAZR imaging system in the following regions of interest (ROI): cancerous tissue (CA); non-cancerous hepatic tissue around the tumors (Peri), and non-cancerous hepatic tissue 2 mm from the tumors (NC). The localization of ICG on the resected specimens' cut surfaces was also evaluated by macroscopic fluorescence imaging with a Maestro imaging system followed by pathological examination with fluorescence microscopy [17].

### Photoacoustic tomography from the body surface in NASH-HCC model mice

Because direct use of the Vevo LAZR imaging system in patients had not yet been approved, PA tomography's ability to visualize hepatic tumors from the body surface was evaluated in a mouse model. Three male NASH-HCC model mice (STAM, Stelic Institute & Co., Tokyo, Japan) were created by administering low-dose streptozotocin after birth (first hit) followed by a subsequent high-fat diet (second hit) [29]. PA tomography was performed on the living 18-week-old NASH-HCC model mice from the body surface to evaluate the detectability of the hepatic tumors. Following PA imaging, all mice were sacrificed, and their livers were screened for HCC nodules by naked-eye examination and fluorescence imaging with the Maestro imaging system.

## Results

### Establishment of PA tomography's ability to visualize ICG-containing cancerous tissue

**Figure 1:**
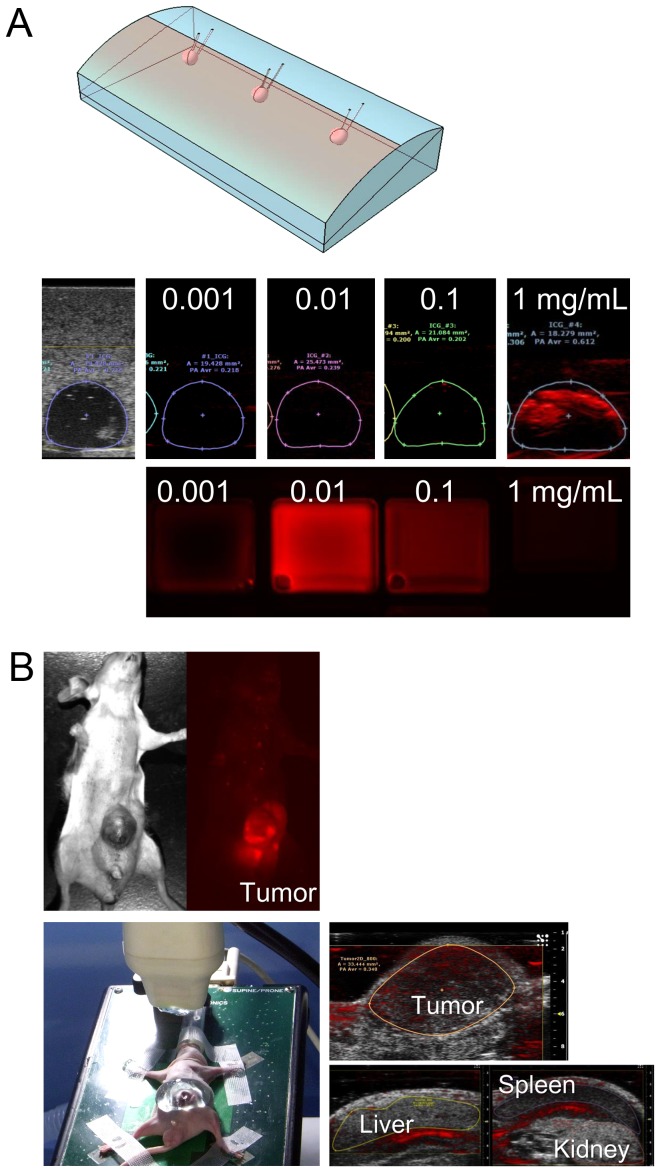
Establishment of PA tomography's ability to visualize ICG-containing tissue. (**A**) Using a human liver tissue-mimicking phantom (top), human plasma containing ICG at concentrations of 0.001, 0.01, 0.1, and 1.0 mg/mL was encapsulated into holes that were 5 mm in diameter and located at a depth of 5 mm from the surface; PA amplitudes were measured using the Vevo LAZR imaging system (middle). Fluorescence images of each ICG-containing plasma sample were also obtained (bottom). (**B**) Fluorescence imaging in a mouse model with subcutaneously implanted well-differentiated human hepatoma cells (HuH-7) identified ICG accumulation in the subcutaneous tumor (left). PA tomography enabled differentiation of tumor-specific PA signals from those of surrounding organs under the conditions of 800-nm excitation light and 54-dB PA gain (right).

For the plasma encapsulated in the tissue-mimicking phantom, at ICG concentrations of 0.001, 0.01, 0.1, and 1.0 mg/mL, the average PA signal intensity under excitation with 800 nm of light was 0.218, 0.239, 0.202, and 0.612, respectively. In contrast, the fluorescence intensity of the ICG-containing plasma increased with increases in ICG concentration up to 0.01 mg/mL, but then started to decrease; this was most likely due to absorption of near-infrared light by ICG (Fig. 1A).

Photoacoustic tomography's ability to identify hepatic malignancies was evaluated in the mice that had undergone subcutaneous implantation of a well-differentiated human hepatoma cell line (HuH-7). Fluorescence imaging at 48 hours after the intravenous injection of ICG demonstrated ICG accumulation in the implanted tumors. Photoacoustic tomography also enabled identification of tumor-specific ICG signals and their visualization on US images under conditions of 800-nm excitation light and 54-dB PA gain (Fig. 1B).

### Photoacoustic tomography of surgically-resected hepatic tissue in humans

**Figure 2:**
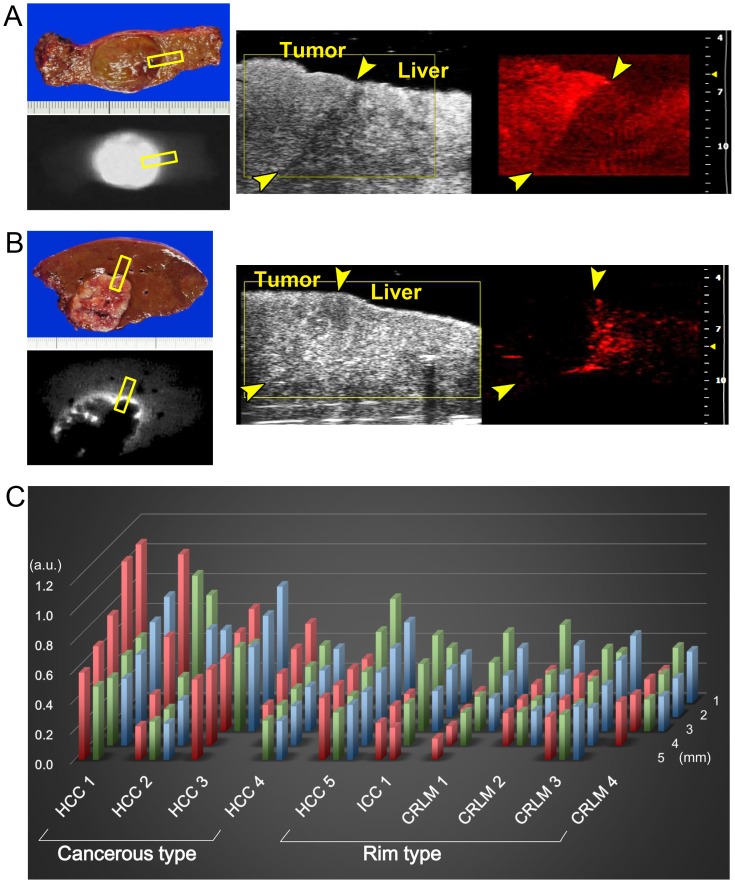
Photoacoustic tomography of surgically-resected hepatic tissue in humans. (**A**) Fluorescence imaging identified uniform fluorescence of ICG on the cut surface of well-differentiated HCC tissue (left). PA tomography from the cut surface of the specimen visualized accumulation of ICG in cancerous tissues on US images (right, please see video S1). (**B**) Fluorescence imaging identified rim-type ICG fluorescence around CRLM lesions on the cut surface of the resected specimen (left). Photoacoustic tomography from the cut surface visualized accumulation of ICG in the peri-cancerous hepatic tissue on US images (right, please see video S2). Yellow squares and arrows in A and B indicate the site where a probe of the imaging system was attached and the boundaries between the tumors and non-cancerous liver parenchyma, respectively. (**C**) Photoacoustic amplitude of each ROI from the resected specimen (red bar indicates cancerous region; green bar, peri-cancerous region; and blue bar, non-cancerous hepatic parenchyma 2 mm from the tumor) according to the depth of the ROI from the sample's surface (depths of 1 to 5 mm). Indocyanine green accumulation was observed in the cancerous tissue in HCC specimens 1–4 (cancerous-type accumulation), while rim-type ICG accumulation was observed in HCC specimen 5, the ICC specimen, and CRLM specimens 1–4.

Table 1 summarizes tumor-related characteristics and the results of fluorescence imaging and PA tomography using ICG. On ICG fluorescence images, 4 out of 5 HCCs showed uniform fluorescence in the cancerous tissue (cancerous-type fluorescence); in the one remaining HCC lesion and all of the adenocarcinoma lesions (one ICC and 4 CRLM lesions), fluorescence of ICG was detected not in the cancerous tissue but rather in the non-cancerous hepatic tissue surrounding the tumors (rim-type fluorescence). Photoacoustic tomography with ICG reproduced these two fluorescence patterns in all 10 resected hepatic specimens (Fig. 2A and B).

**Table 1:** Tumor characteristics and results of fluorescence and PA imaging.

Tumors	Age(y)/Sex	ICG R15 (%)	Interval between ICG injection and surgery (d)	CH/LC	Cancer cell differentiation	Tumor diameter (mm)	Fluorescence patterns	PA patterns
HCC 1	76/F	19.6	40	Yes	Well	24	Cancerous	Cancerous
HCC 2	85/M	1.8	2	No	Moderate	97	Cancerous	Cancerous
HCC 3	69/M	9.8	29	Yes	Moderate	27	Cancerous	Cancerous
HCC 4	80/M	20.7	10	No	Moderate	36	Cancerous	Cancerous
HCC 5	73/F	7.4	14	No	Moderate	23	Rim	Rim
ICC 1	70/F	8.6	8	No	Moderate	59	Rim	Rim
CRLM 1	54/M	9.4	27	No	tub2	15	Rim	Rim
CRLM 2	54/M	9.4	27	No	tub2	16	Rim	Rim
CRLM 3	84/M	8.5	38	No	tub1	20	Rim	Rim
CRLM 4	53/M	1.9	24	No	tub2	17	Rim	Rim

Abbreviations: PA imaging, photoacoustic imaging; ICG R15, preoperative indocyanine green retention rate at 15 minutes; CH/LC, presence of chronic hepatitis or liver cirrhosis; HCC, hepatocellular carcinoma; ICC, intrahepatic cholangiocarcinoma; CRLM, colorectal liver metastasis.

Photoacoustic signal amplitude according to ROI (CA, Peri, and NC) is demonstrated in Fig. 2C. In the CA tissue of HCCs that showed cancerous-type fluorescence and in the Peri tissue of tumors that showed rim-type fluorescence, PA signal amplitude decreased with increases in distance between the liver surface and the ROI: at 4 mm deep to the liver surface, the PA amplitudes ranged from 43% to 83% (median, 61%) of those measured just beneath the liver surface (depth of 1 mm). Thus, ROIs within 3 mm of the liver surface were used for the following analysis of the resected liver specimens to minimize the effect of attenuation on the PA signals.

The ratio of PA signal amplitude in CA tissue relative to that in NC tissue was higher in the 4 tumors showing cancerous-type ICG fluorescence compared with the 6 tumors with rim-type fluorescence (median [range], 1.5 [0.8–1.8] vs. 0.7 [0.5–0.8], *P* = 0.01 [Wilcoxon's rank-sum test]). In contrast, the ratio of PA signal amplitudes in Peri tissue relative to that in NC tissue was lower in the former group.

**Figure 3:**
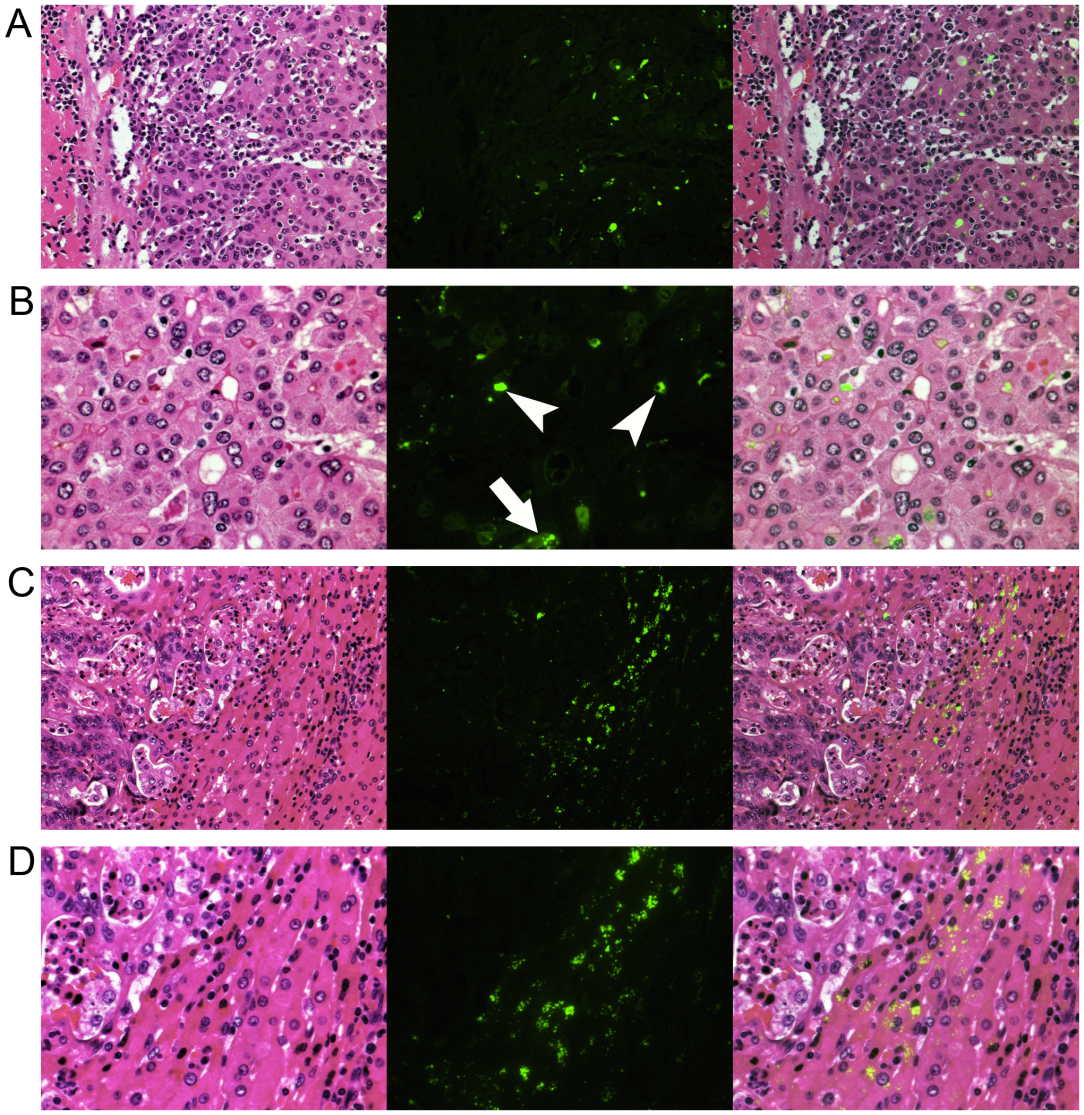
Fluorescence microscopy. Left, hematoxylin-eosin staining; middle, fluorescence images; and right, fusion images of ICG fluorescence, indicated in green, and hematoxylin-eosin staining. (**A**) In well-differentiated HC lesions, ICG fluorescence was identified mainly in the cancerous tissue, as demonstrated in Figure 2A. (**B**) Magnified view of (A). Indocyanine green had accumulated in the pseudoglands (arrowheads) and the cytoplasm of cancer cells (arrow). (**C**) Indocyanine green fluorescence was identified in the peri-cancerous hepatic parenchyma surrounding a CRLM lesion, as demonstrated in Figure 2B. (**D**) Magnified view of (C). Indocyanine green had accumulated in the cytoplasm of relatively small hepatocytes rather than in the intracellular spaces.

Fluorescent microscopy revealed the presence of ICG in pseudoglands and cancer cell cytoplasm from HCC tissue showing cancerous-type fluorescence and PA signals (Fig. 3A and B). In contrast, in hepatic tumors showing rim-type fluorescence and PA signals, ICG was identified mainly in the cytoplasm of the hepatocytes surrounding the tumors (Fig. 3C and D).

### Photoacoustic tomography from the body surface in NASH-HCC model mice

**Figure 4:**
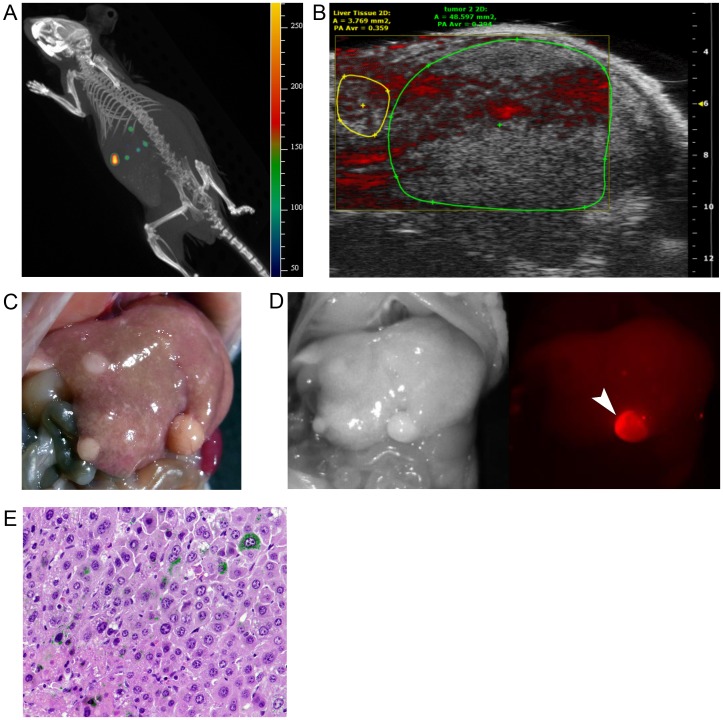
Photoacoustic tomography from the body surface in NASH-HCC model mice. (**A**) Spectrum computed tomography of a living NASH-HCC model mouse identified hepatic tumors with accumulation of ICG that had been intravenously injected 48 hours prior (IVIS Spectrum CT, PerkinElmer, Hopkinton, US; excitation 745 nm, emission 800 nm). (**B**) Photoacoustic tomography failed to visualize any cancer-specific PA signals on US images. (**C, D**) Three liver nodules were macroscopically identified in this mouse (C); one was visualized on fluorescence imaging (D). (**E**) Fluorescence microscopy identified ICG fluorescence (demonstrated in green in this fusion image with hematoxylin-eosin staining) in the cytoplasm of some of the cancerous cells in this NASH-HCC model mouse.

In the three NASH-HCC model mice, PA tomography was applied to assess hepatic tumor detection from the body surface. Despite the identification of cancerous nodules on US images and spectrum computed tomography, PA tomography was unable to visualize any of these nodules with sufficient signal contrast relative to the background hepatic parenchyma (Fig. 4A and B). On abdominal exploration following sacrifice of the mice, a total of 10 hepatic tumors were macroscopically identified and then microscopically proven to be HCCs that had developed in the NASH livers. Among the 10 HCCs, 4 tumors were visualized by fluorescence imaging of the liver surface (Fig. 4C and D). Fluorescence microscopy demonstrated ICG accumulation in the cytoplasm of cancerous cells, but ICG-positive cells were infrequent compared with their occurrence in human HCC tissue.

## Discussion

In the surgical treatment of hepatic malignancies, US examination has played an indispensable role in the preoperative identification and differential diagnosis of hepatic lesions [30], [31]. Recently, intraoperative fluorescence imaging using ICG has begun to be used to detect hepatic tumors on the liver surface prior to hepatectomy and to estimate the histological diagnosis for resected hepatic tumors [12]–[17]. In the present study, PA tomography enabled clear visualization of the distribution of preoperatively-administered ICG in and/or around hepatic tumors on US images of the surgically-resected specimens. Our results suggest that PA imaging with ICG has the potential to develop into a novel diagnostic tool that can support the localization and differential diagnosis of hepatic tumors established by conventional US examination and intraoperative fluorescence imaging; it does this by enabling the visualization of biliary excretory function in hepatic tumors and non-cancerous hepatic parenchyma.

In the present series, all but one HCC showed cancerous-type ICG fluorescence on the cut surfaces of the resected specimens, while all of the CRLM lesions and the ICC demonstrated rim-type fluorescence. These patterns of ICG accumulation were well reproduced by PA tomography performed from the surface of the resected specimens. In a previous study, the mechanism of cancerous-type fluorescence in HCC was revealed [17]: differentiated HCC cells maintain portal uptake function, but because of biliary excretion disorders caused by functional and/or morphological changes (as suggested by fluorescence microscopy in the present study), ICG accumulates in cancerous tissues. In contrast, ICG accumulation in the non-cancerous hepatic parenchyma around adenocarcinoma lesions has not yet been fully explained. It may be partly due to local biliary congestion resulting from simple compression by the tumors; however, a recent study by van der Vorst and colleagues [15] suggested that the increased presence of immature hepatocytes along with the impaired expression of organic anion transporters could also cause ICG accumulation in peri-cancerous hepatic regions. This theory is supported by the microscopic findings in the present study, in which ICG fluorescence was primarily detected in the cytoplasm of relatively small hepatocytes rather than in the intercellular spaces around these tumors. Visualization of hepatic ICG distribution by PA tomography may be useful not only in the differential diagnosis of hepatic tumors during conventional US examination (by assessing the tumors' bile-producing ability) but also for monitoring the delivery of anticancer agents/sensitizers with biliary excretion properties used for chemotherapy, radiotherapy, and photodynamic treatment.

The major limitation of the current technique used for PA tomography with ICG lies in the small region observable from the liver surface. Although PA signals can be detected from depths of up to 7 cm in theory [32], in our study of resected human hepatic specimens, PA amplitudes markedly decreased with depth of the ROI from the liver surface, decreasing by approximately 40% even at 4 mm in depth. In the mouse models, PA tomography with ICG failed to identify any of the HCC nodules that had developed in the NASH livers. These problems with PA tomography can be primarily attributed to steep attenuation of the laser pulses used for excitation of ICG and/or attenuation of the PA signals emitted from the targeted regions; these issues can be solved, at least in part, by improving the intensity of the pulse laser and the sensitivity of the detector used in PA imaging systems. Another possible cause of the limited observable range identified in the present study is that the amount of hepatic tumor ICG accumulation was optimal for fluorescence imaging but insufficient for PA tomography, especially in the mouse model, where imaging was performed from the body surface. In the tissue-mimicking phantom plasma samples, PA imaging required at least 10 times the ICG concentration needed for fluorescence imaging, but such a high dosage of intravenous injection of ICG is not realistic (LD_50_ value of ICG is 64.3–72.8 mg/kg in mice) [33]. Novel contrast materials, such as metallic nanoparticles [26] and carbon nanotubes [34], may enable clear visualization of PA signals emitted from deeply-located hepatic tumors through biliary excretion disorders in and around cancerous tissues, if these agents have biliary excretion properties similar to ICG.

In conclusion, PA tomography using ICG is a promising technique that can support US examination of the liver by providing information on biliary excretion disorders in cancerous tissues and peri-cancerous hepatic parenchyma. However, further technical improvement is needed to enable the visualization of deeply-located lesions.

## Supporting Information

Video S1 **Three-dimensional reconstruction of PA tomography from a human hepatic specimen containing well-differentiated HCC.**
(WMV)

Video S2 **Three-dimensional reconstruction of PA tomography from a human hepatic specimen containing CRLM.**
(WMV)

Data S1 **Raw data of the present study.**
(ZIP)

## References

[1] Ogata F, Azuma R, Kikuchi M, Koshima I, Morimoto Y (2007) Novel lymphography using indocyanine green dye for near-infrared fluorescence labeling. Ann Plast Surg 58: 652–655. 17522489 10.1097/01.sap.0000250896.42800.a2

[2] Kitai T, Inomoto T, Miwa M, Shikayama T (2005) Fluorescence navigation with indocyanine green for detecting sentinel lymph nodes in breast cancer. Breast Cancer 12: 211–215. 16110291 10.2325/jbcs.12.211

[3] Mitsuhashi N, Kimura F, Shimizu H, Imamaki M, Yoshidome H, et al (2008) Usefulness of intraoperative fluorescence imaging to evaluate local anatomy in hepatobiliary surgery. J Hepatobiliary Pancreat Surg 15: 508–514. 18836805 10.1007/s00534-007-1307-5

[4] Ishizawa T, Tamura S, Masuda K, Aoki T, Hasegawa K, et al (2009) Intraoperative fluorescent cholangiography using indocyanine green: a biliary road map for safe surgery. J Am Coll Surg 208: e1–e4. 10.1016/j.jamcollsurg.2008.09.024 19228492

[5] Ishizawa T, Bandai Y, Ijichi M, Kaneko J, Hasegawa K, et al (2010) Fluorescent cholangiography illuminating the biliary tree during laparoscopic cholecystectomy. Br J Surg 97: 1369–1377. 20623766 10.1002/bjs.7125

[6] Schols RM, Bouvy ND, Masclee AA, van Dam RM, Dejong CH, et al (2013) Fluorescence cholangiography during laparoscopic cholecystectomy: a feasibility study on early biliary tract delineation. Surg Endosc 2013 27: 1530–1536. 10.1007/s00464-012-2635-3 23076461

[7] Spinoglio G, Priora F, Bianchi PP, Lucido FS, Licciardello A, et al (2013) Real-time near-infrared (NIR) fluorescent cholangiography in single-site robotic cholecystectomy (SSRC): a single-institutional prospective study. Surg Endosc 27: 2156–2162. 23271272 10.1007/s00464-012-2733-2

[8] Rubens FD, Ruel M, Fremes SE (2002) A new and simplified method for coronary and graft imaging during CABG. Heart Surg Forum 5: 141–144. 12114127

[9] Raabe A, Nakaji P, Beck J, Kim LJ, Hsu FP, et al (2005) Prospective evaluation of surgical microscope-integrated intraoperative near-infrared indocyanine green videoangiography during aneurysm surgery. J Neurosurg 103: 982–989. 16381184 10.3171/jns.2005.103.6.0982

[10] Kawaguchi Y, Ishizawa T, Miyata Y, Yamashita S, Masuda K, et al (2013) Portal uptake function in veno-occlusive regions evaluated by real-time fluorescent imaging using indocyanine green. J Hepatol 58: 247–253. 23041306 10.1016/j.jhep.2012.09.028

[11] Ris F, Hompes R, Cunningham C, Lindsey I, Guy R, et al (2014) Near-infrared (NIR) perfusion angiography in minimally invasive colorectal surgery. Surg Endosc 28: 2221–2226. 24566744 10.1007/s00464-014-3432-y PMC4065377

[12] Ishizawa T, Fukushima N, Shibahara J, Masuda K, Tamura S, et al (2009) Real-time identification of liver cancers by using indocyanine green fluorescent imaging. Cancer 115: 2491–2504. 19326450 10.1002/cncr.24291

[13] Gotoh K, Yamada T, Ishikawa O, Takahashi H, Eguchi H, et al (2009) A novel image-guided surgery of hepatocellular carcinoma by indocyanine green fluorescence imaging navigation. J Surg Oncol 100: 75–79. 19301311 10.1002/jso.21272

[14] Yokoyama N, Otani T, Hashidate H, Maeda C, Katada T, et al (2012) Real-time detection of hepatic micrometastases from pancreatic cancer by intraoperative fluorescence imaging: Preliminary results of a prospective study. Cancer 118: 2813–2819. 21990070 10.1002/cncr.26594

[15] van der Vorst JR, Schaafsma BE, Hutteman M, Verbeek FP, Liefers GJ, et al (2013) Near-infrared fluorescence-guided resection of colorectal liver metastases. Cancer 119: 3411–3418. 23794086 10.1002/cncr.28203 PMC3775857

[16] Kudo H, Ishizawa T, Tani K, Harada N, Ichida A, et al (2014) Visualization of subcapsular hepatic malignancy by indocyanine-green fluorescence imaging during laparoscopic hepatectomy. Surg Endosc 28: 2504–2508. 24566751 10.1007/s00464-014-3468-z

[17] Ishizawa T, Masuda K, Urano Y, Kawaguchi Y, Satou S, et al (2014) Mechanistic background and clinical applications of indocyanine green fluorescence imaging of hepatocellular carcinoma. Ann Surg Oncol 21: 440–448. 24254203 10.1245/s10434-013-3360-4

[18] Kruger RA (1994) Photoacoustic ultrasound. Med Phys 21: 127–131. 8164577 10.1118/1.597367

[19] Oraevsky AA, Jacques SL, Tittel FK (1997) Measurement of tissue optical properties by time-resolved detection of laser-induced transient stress. Appl Opt 36: 402–415. 18250688 10.1364/ao.36.000402

[20] Wang LV (2009) Multiscale photoacoustic microscopy and computed tomography. Nat Photonics 29: 503–509. 10.1038/nphoton.2009.157 PMC2802217 20161535

[21] Kim C, Song KH, Gao F, Wang LV (2010) Sentinel lymph nodes and lymphatic vessels: noninvasive dual-modality in vivo mapping by using indocyanine green in rats–volumetric spectroscopic photoacoustic imaging and planar fluorescence imaging. Radiology 255: 442–450. 20413757 10.1148/radiol.10090281 PMC2858815

[22] Rajian JR, Fabiilli ML, Fowlkes JB, Carson PL, Wang X (2011) Drug delivery monitoring by photoacoustic tomography with an ICG encapsulated double emulsion. Opt Express 19: 14335–14347. 21934797 10.1364/OE.19.014335 PMC3324934

[23] Taruttis A, Morscher S, Burton NC, Razansky D, Ntziachristos V (2012) Fast multispectral optoacoustic tomography (MSOT) for dynamic imaging of pharmacokinetics and biodistribution in multiple organs. PLoS One 7: 30491. 10.1371/journal.pone.0030491 PMC3266258 22295087

[24] Chino K, Akagi R, Dohi M, Fukashiro S, Takahashi H (2012) Reliability and validity of quantifying absolute muscle hardness using ultrasound elastography. PLoS One 7: e45764. 23029231 10.1371/journal.pone.0045764 PMC3448710

[25] Needles A, Heinmiller A, Sun J, Theodoropoulos C, Bates D, et al (2013) Development and initial application of a fully integrated photoacoustic micro-ultrasound system. IEEE Trans Ultrason Ferroelectr Freq Control 60: 888–897. 23661123 10.1109/TUFFC.2013.2646

[26] Nam SY, Ricles LM, Suggs LJ, Emelianov SY (2012) In vivo Ultrasound and Photoacoustic Monitoring of Mesenchymal Stem Cells Labeled with Gold Nanotracers. PLoS One 7: e37267. 22615959 10.1371/journal.pone.0037267 PMC3353925

[27] Nakabayashi H, Taketa K, Miyano K, Yamane T, Sato J (1982) Growth of human hepatoma cells lines with differentiated functions in chemically defined medium. Cancer Res 42: 3858–3863. 6286115

[28] Kaneko J, Inagaki Y, Ishizawa T, Gao J, Tang W, et al (2014) Photodynamic therapy for human hepatoma-cell-line tumors utilizing biliary excretion properties of indocyanine green. J Gastroenterol 49: 110–116. 23595610 10.1007/s00535-013-0775-4

[29] Fujii M, Shibazaki Y, Wakamatsu K, Honda Y, Kawauchi Y, et al (2013) A murine model for non-alcoholic steatohepatitis showing evidence of association between diabetes and hepatocellular carcinoma. Med Mol Morphol 46: 141–152. 23430399 10.1007/s00795-013-0016-1

[30] Zhang K, Kokudo N, Hasegawa K, Arita J, Tang W, et al (2007) Detection of new tumors by intraoperative ultrasonography during repeated hepatic resections for hepatocellular carcinoma. Arch Surg 142: 1170–1175. 18086983 10.1001/archsurg.142.12.1170

[31] Takahashi M, Hasegawa K, Arita J, Hata S, Aoki T, et al (2012) Contrast-enhanced intraoperative ultrasonography using perfluorobutane microbubbles for the enumeration of colorectal liver metastases. Br J Surg 99: 1271–1277. 22829436 10.1002/bjs.8844

[32] Wang LV, Hu S (2012) Photoacoustic tomography: in vivo imaging from organelles to organs. Science 335: 1458–1462. 22442475 10.1126/science.1216210 PMC3322413

[33] Drug interview form of diagnogreen for injection. Available: https://www.medicallibrary-dsc.info/di/diagnogreen_for_injection_25mg/pdf/if_dg_inj_1305_08.pdf. Accessed 2013 May 01.

[34] De la Zerda A, Zavaleta C, Keren S, Vaithilingam S, Bodapati S, et al (2008) Carbon nanotubes as photoacoustic molecular imaging agents in living mice. Nat Nanotechnol 3: 557–562. 18772918 10.1038/nnano.2008.231 PMC2562547

