# Supplementary material for: Photoacoustic Tomography of Human Hepatic Malignancies Using Intraoperative Indocyanine Green Fluorescence Imaging
Source: PLoS One. 2014 Nov 7;9(11):e112667. doi: 10.1371/journal.pone.0112667 (PMC4224503; doi:10.1371/journal.pone.0112667)
Supplement: Data S1 — Raw data of the present study. (ZIP) [file pone.0112667.s003.zip › SupplementaryMaterials_Ishizawa/Images_Maestro.pptx]

## Slide 1
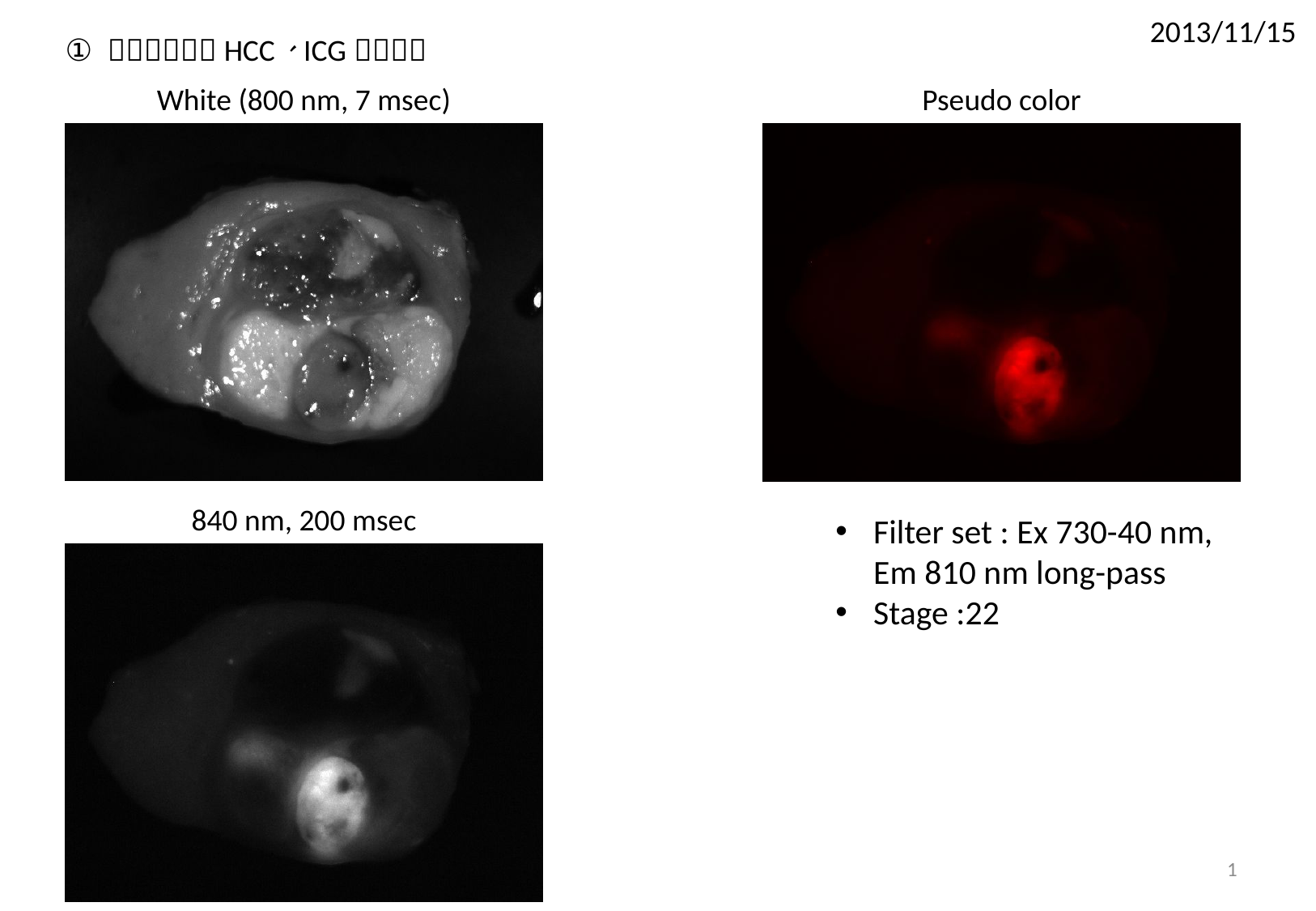

2013/11/15
① ヒトサンプルHCC、ICG注射済み
White (800 nm, 7 msec)
Pseudo color
840 nm, 200 msec
Filter set : Ex 730-40 nm, Em 810 nm long-pass
Stage :22
1

## Slide 2
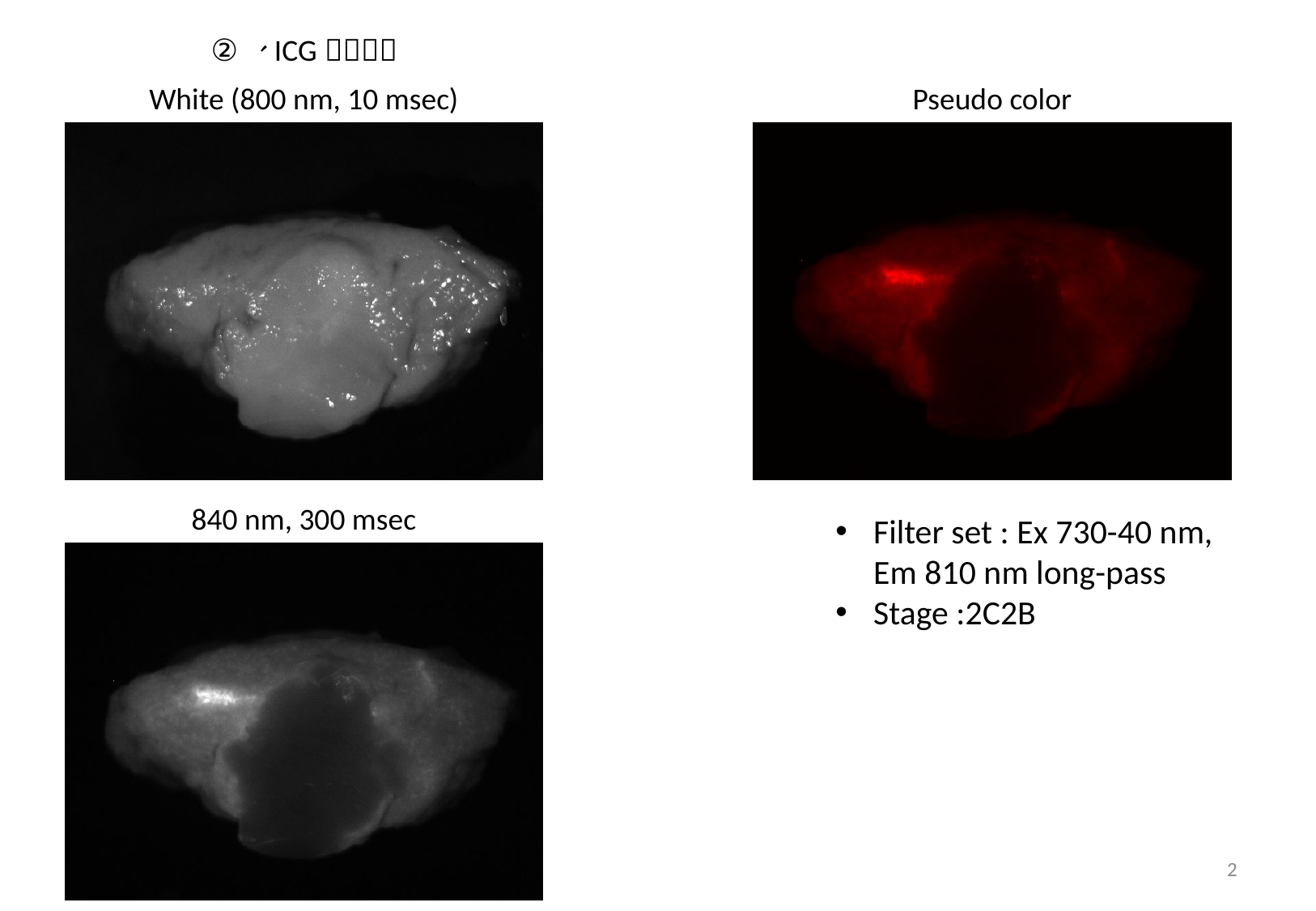

② ヒトサンプル（大腸肝メタ）、ICG注射済み
White (800 nm, 10 msec)
Pseudo color
840 nm, 300 msec
Filter set : Ex 730-40 nm, Em 810 nm long-pass
Stage :2C2B
2

## Slide 3
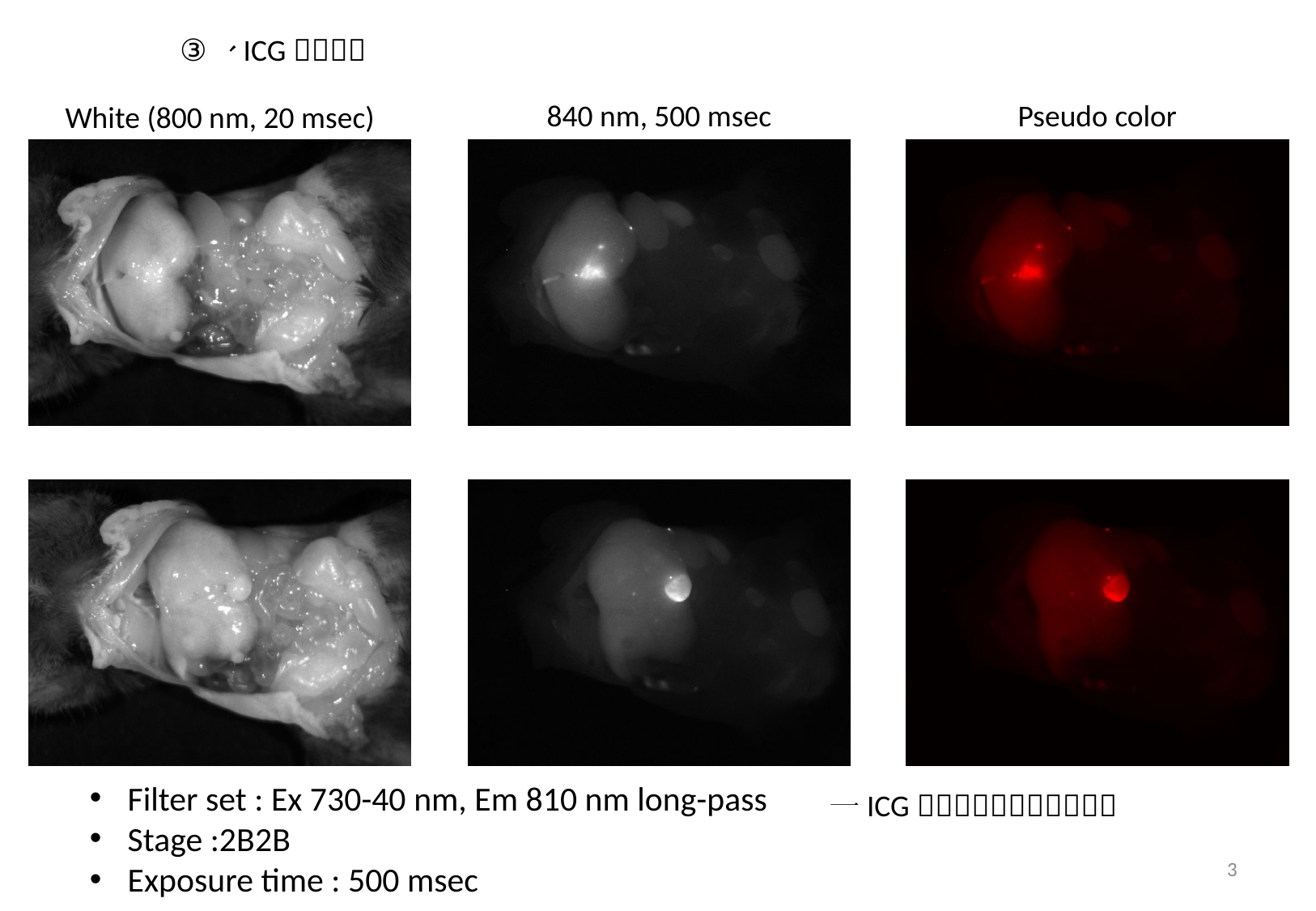

③ 脂肪肝モデルマウス、ICG注射済み
840 nm, 500 msec
Pseudo color
White (800 nm, 20 msec)
Filter set : Ex 730-40 nm, Em 810 nm long-pass
Stage :2B2B
Exposure time : 500 msec
一部の腫瘍（大きい物４つ中２つ）のみからICG由来の蛍光が検出された
3

## Slide 4
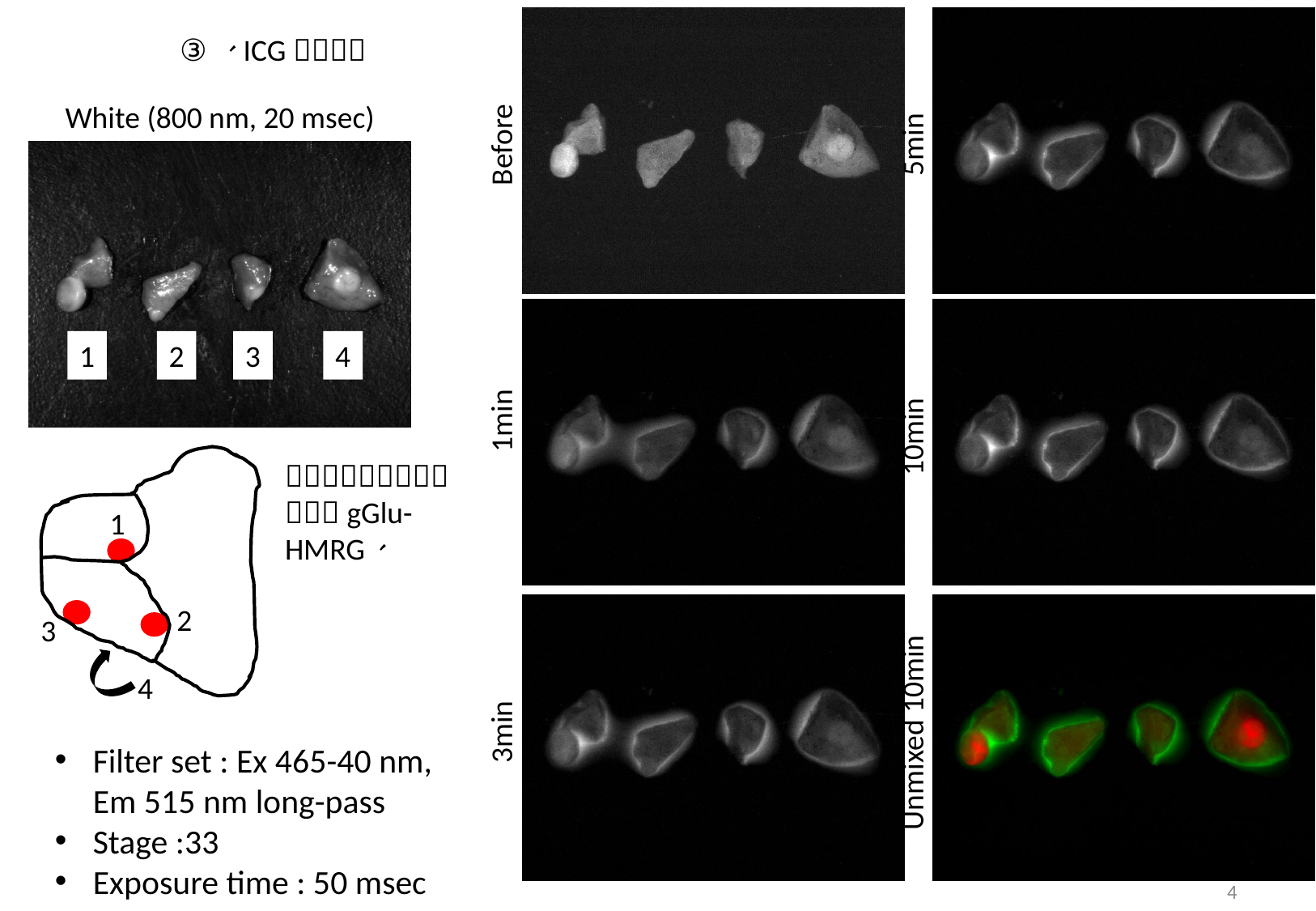

③ 脂肪肝モデルマウス、ICG注射済み
White (800 nm, 20 msec)
Before
5min
1
2
3
4
1min
10min
1
2
3
4
大きい腫瘍を切除後さらにgGlu-HMRGを滴下してイメージングしたが、光るものはなかった
Unmixed 10min
3min
Filter set : Ex 465-40 nm, Em 515 nm long-pass
Stage :33
Exposure time : 50 msec
4

## Slide 5
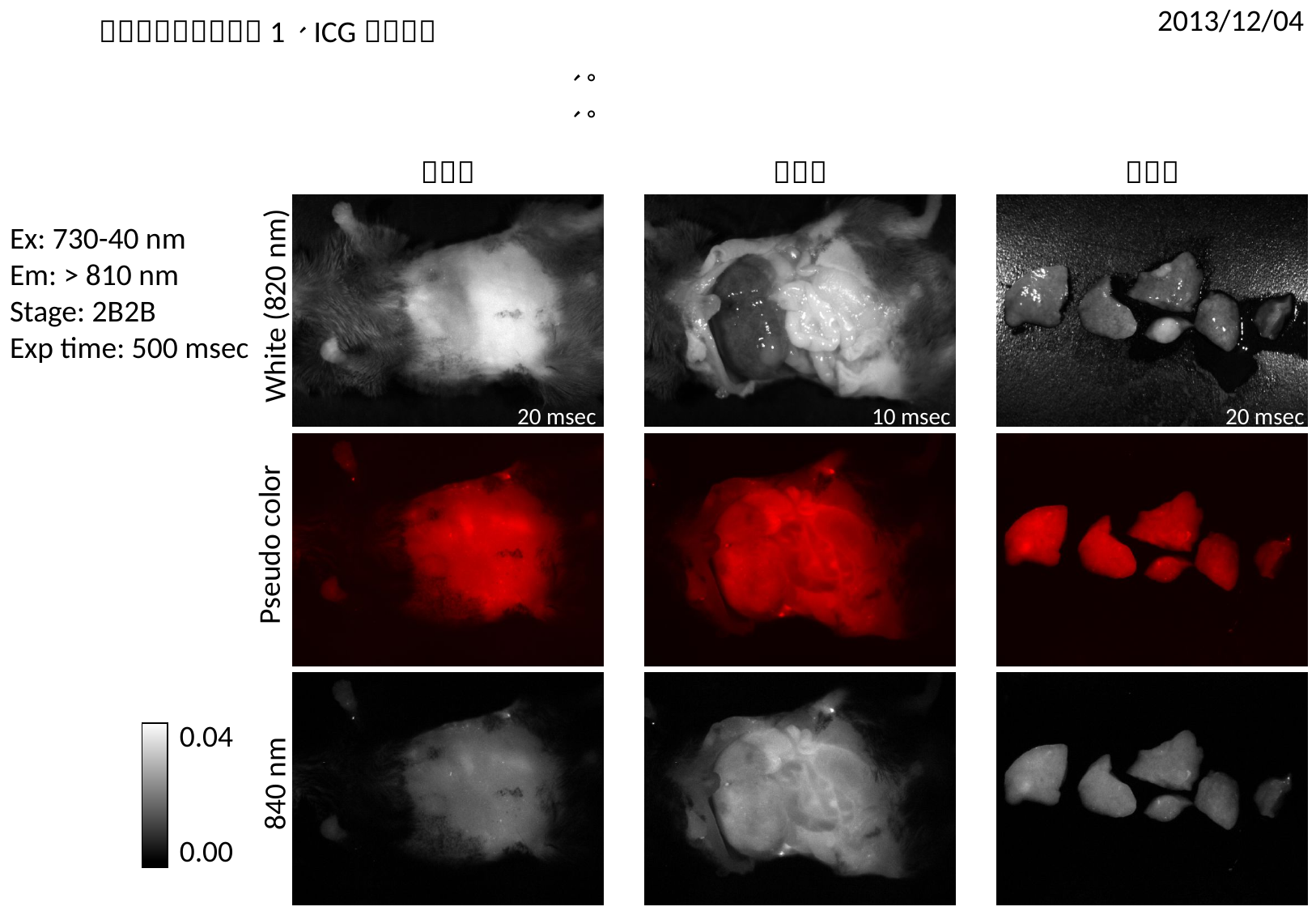

2013/12/04
脂肪肝モデルマウス1匹、ICG注射済み
開腹前、開腹後ともに蛍光は観察できなかった。
肝臓にあった腫瘤様な部分に割を入れてイメージングしても、蛍光は観察できなかった。
開腹前
開腹後
摘出後
White (820 nm)
Ex: 730-40 nm
Em: > 810 nm
Stage: 2B2B
Exp time: 500 msec
20 msec
10 msec
20 msec
Pseudo color
0.04
0.00
840 nm

## Slide 6
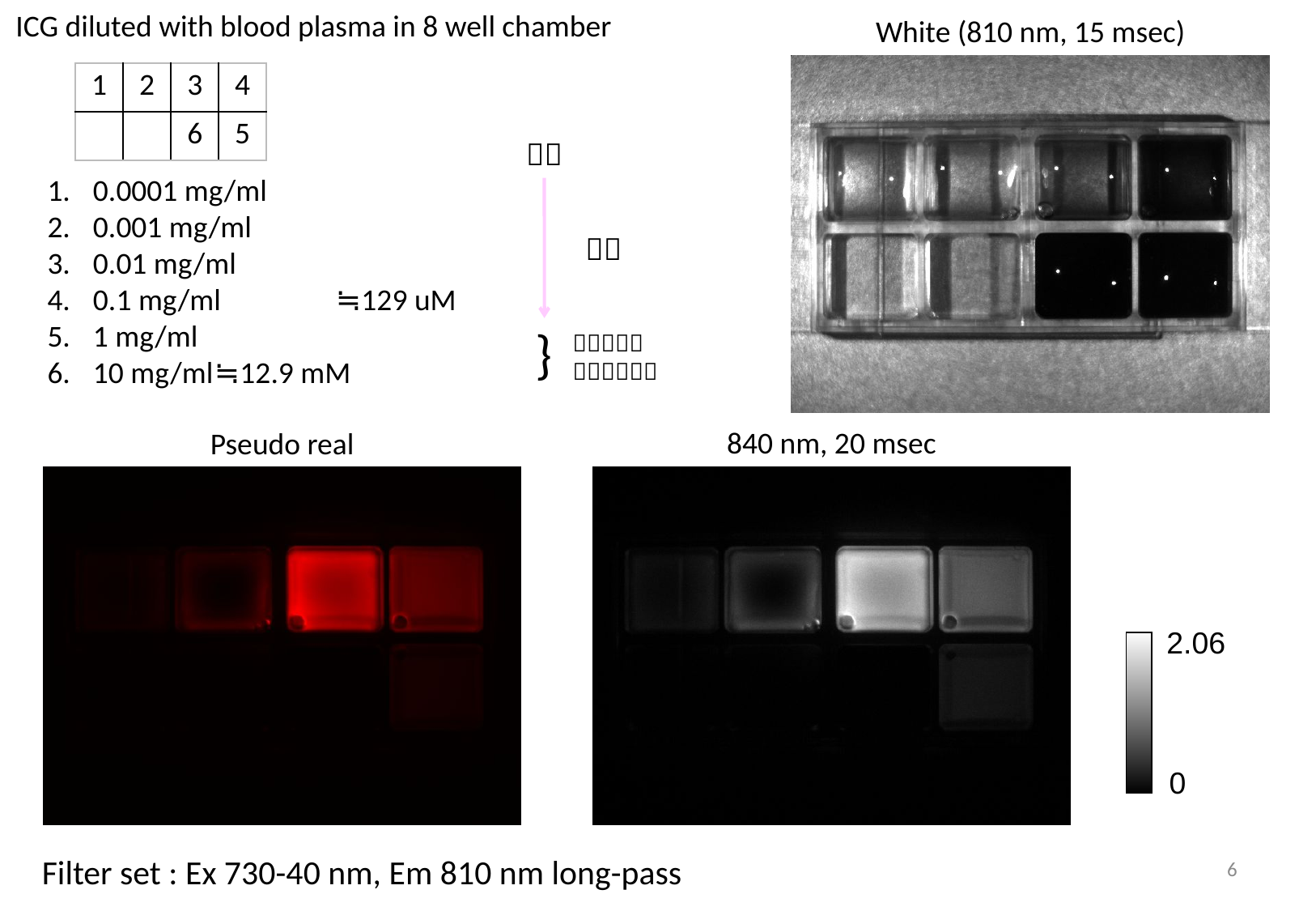

ICG diluted with blood plasma in 8 well chamber
White (810 nm, 15 msec)
| 1 | 2 | 3 | 4 |
| --- | --- | --- | --- |
| | | 6 | 5 |
蛍光
0.0001 mg/ml
0.001 mg/ml
0.01 mg/ml
0.1 mg/ml	≒129 uM
1 mg/ml
10 mg/ml	≒12.9 mM
上昇
}
濃度消光で
見えなくなる
840 nm, 20 msec
Pseudo real
2.06
0
6
Filter set : Ex 730-40 nm, Em 810 nm long-pass

## Slide 7
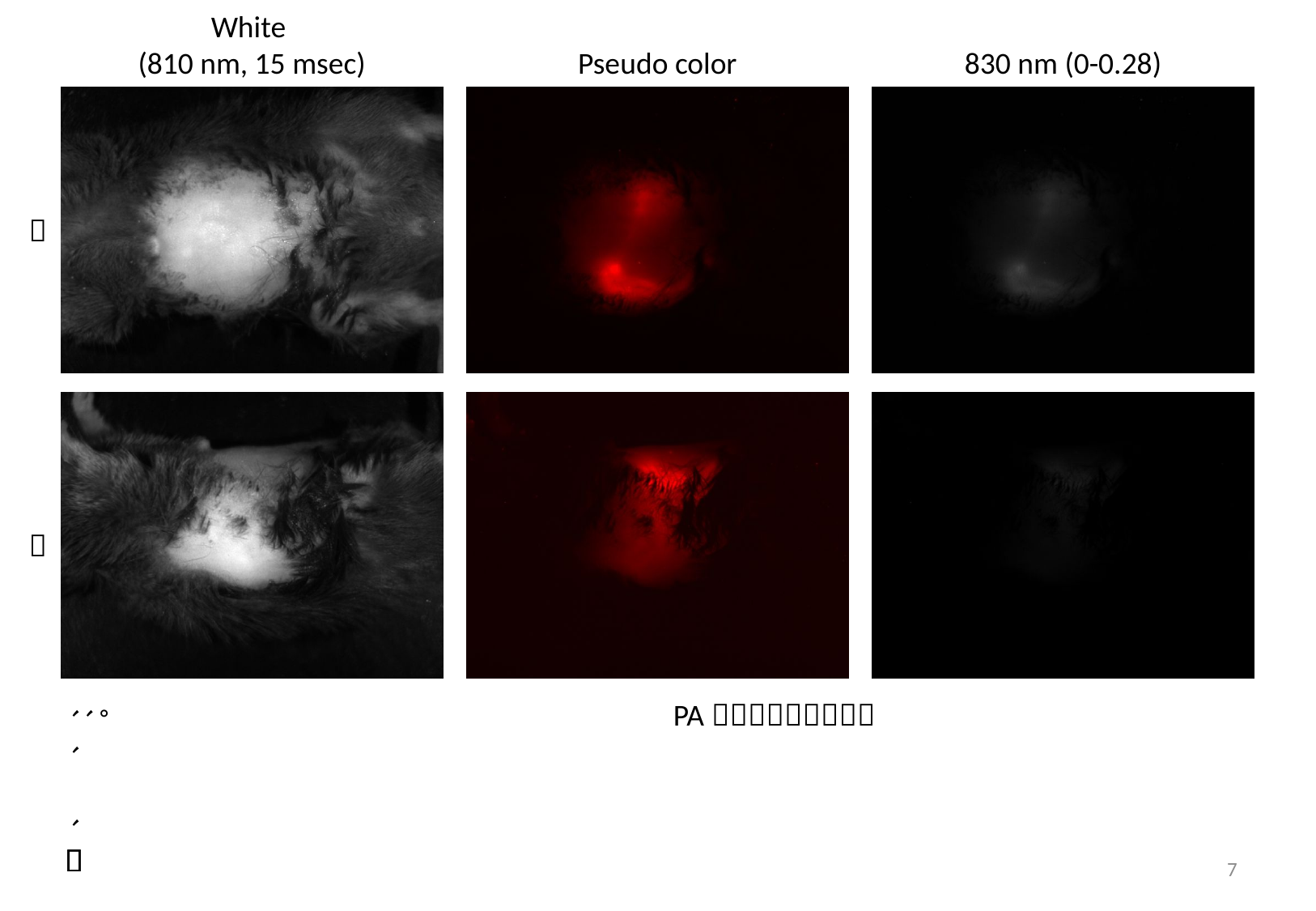

White
(810 nm, 15 msec)
Pseudo color
830 nm (0-0.28)
１
２
マウスを安楽殺後、心臓から脱血し、開腹前にイメージングを行った。（PA実験時に脱毛済み）
１：伏臥位、２：左側臥位
いずれも腫瘍と思われる部分からの蛍光は観察できず、腸由来と考えられる非常に弱い
蛍光のみが観察された。
7

## Slide 8
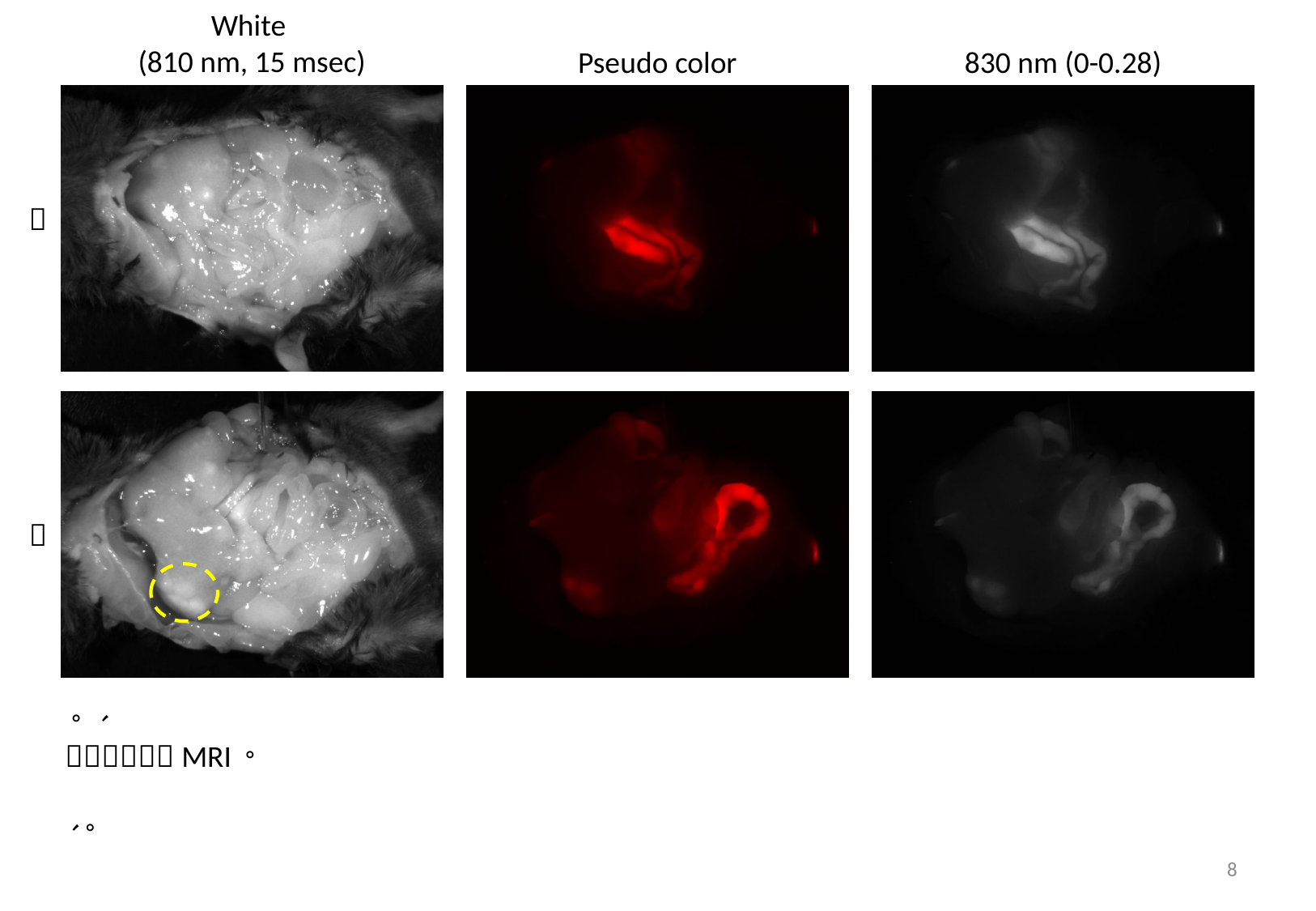

White
(810 nm, 15 msec)
Pseudo color
830 nm (0-0.28)
３
４
開腹後の像。３：開腹直後、４：黄円で囲んだ腫瘍が見やすいように腸管をずらした状態
黄円の腫瘍がMRIで背側に見えた最大の腫瘍だと考えられる。
いずれも腫瘍と思われる部分からの蛍光は弱く、腸管から比較的強い蛍光が見られる。
8

## Slide 9
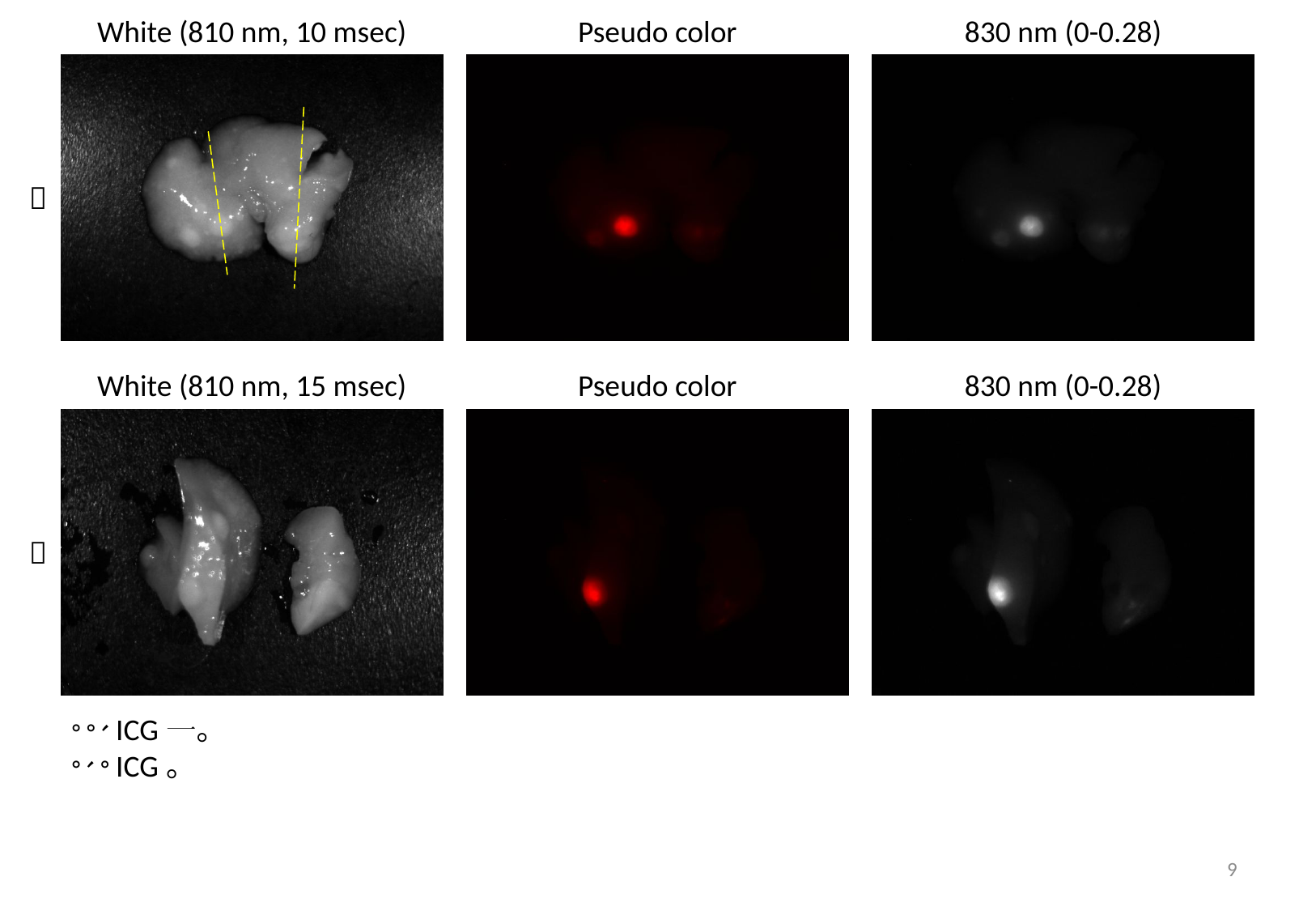

White (810 nm, 10 msec)
Pseudo color
830 nm (0-0.28)
５
White (810 nm, 15 msec)
Pseudo color
830 nm (0-0.28)
６
５：肝臓を摘出しイメージング。３や４では肋骨に隠れていた比較的小さい腫瘍から最大の蛍光を観察。他の腫瘍から弱い蛍光しか観察できなかったことから、ICGの集積は腫瘍間で一定ではないことがわかる。
６：黄線の場所で割を入れてイメージング。左が最大蛍光を示した腫瘍、右が４で黄円で示した最も大きな腫瘍。ICGは腫瘍の中まで到達していることがわかる。
9

## Slide 10
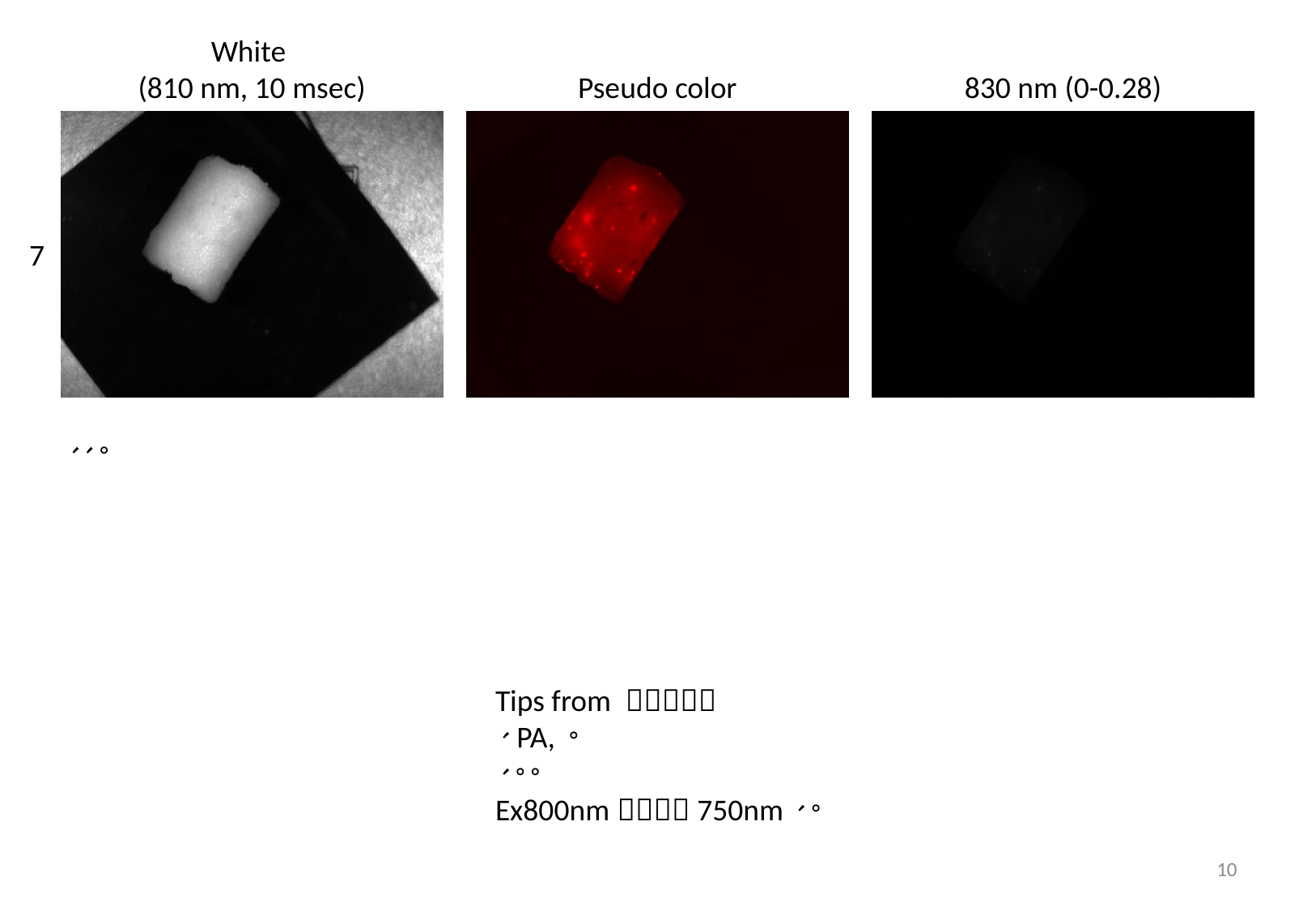

White
(810 nm, 10 msec)
Pseudo color
830 nm (0-0.28)
7
当モデルマウスの飼育に用いられている飼料は特殊飼料であるため、自家蛍光の有無を確認したが、今回使用したフィルターセット下では蛍光はほとんど観察できなかった。
Tips from 水流さん：
超音波検査用ジェルに泡が入ると、PA,エコー共に画像が乱れる。
脈管が光っていて、がんで光って見えないことあり。がんのフチが見えたことはある。
Ex800nmのほうが750nmに比べて、シグナル総量は少ないがコントラストがよくつく。
10

## Slide 11
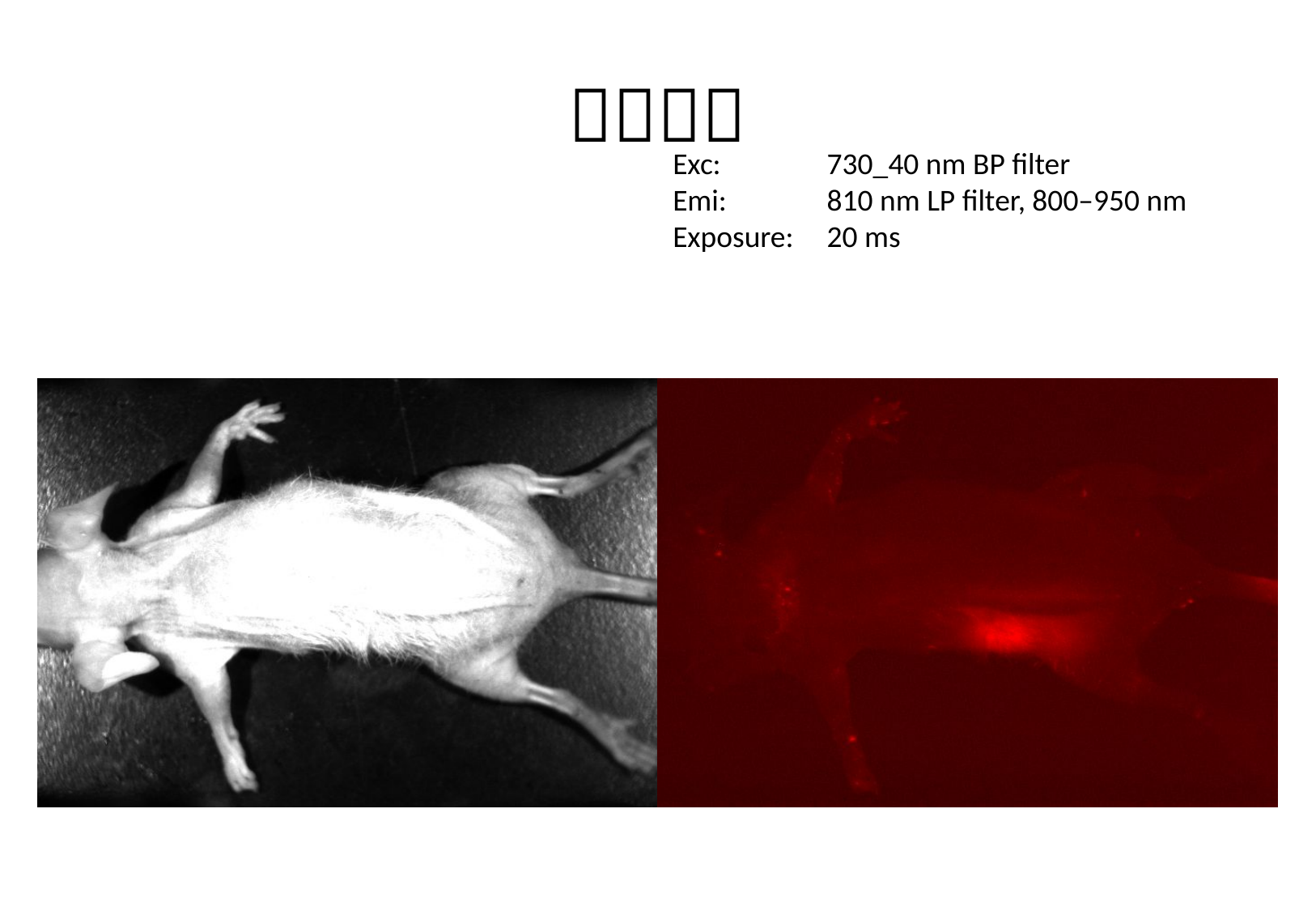

# 開腹前１
Exc: 	730_40 nm BP filter
Emi: 	810 nm LP filter, 800–950 nm
Exposure: 	20 ms

## Slide 12
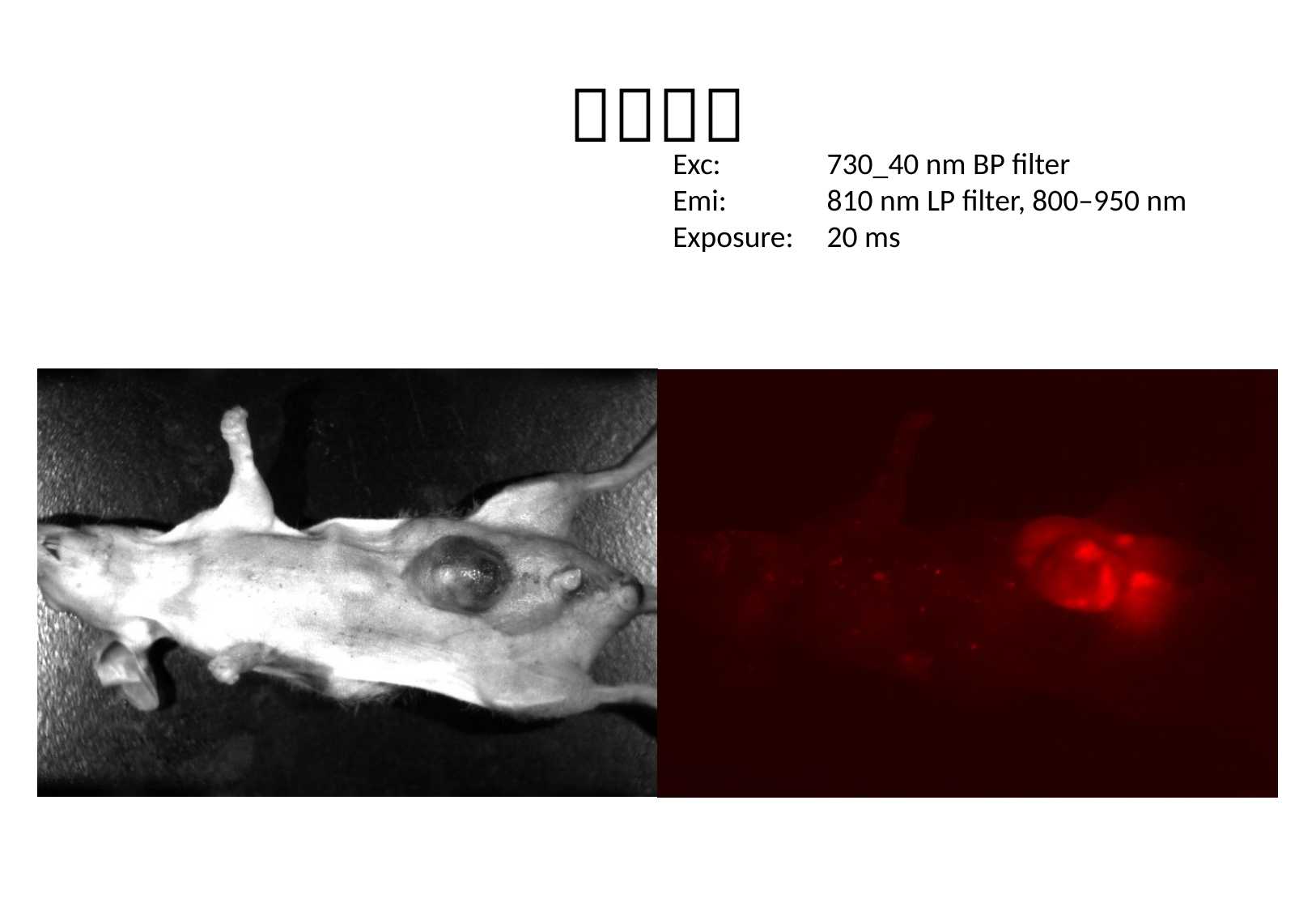

# 開腹前２
Exc: 	730_40 nm BP filter
Emi: 	810 nm LP filter, 800–950 nm
Exposure: 	20 ms

## Slide 13
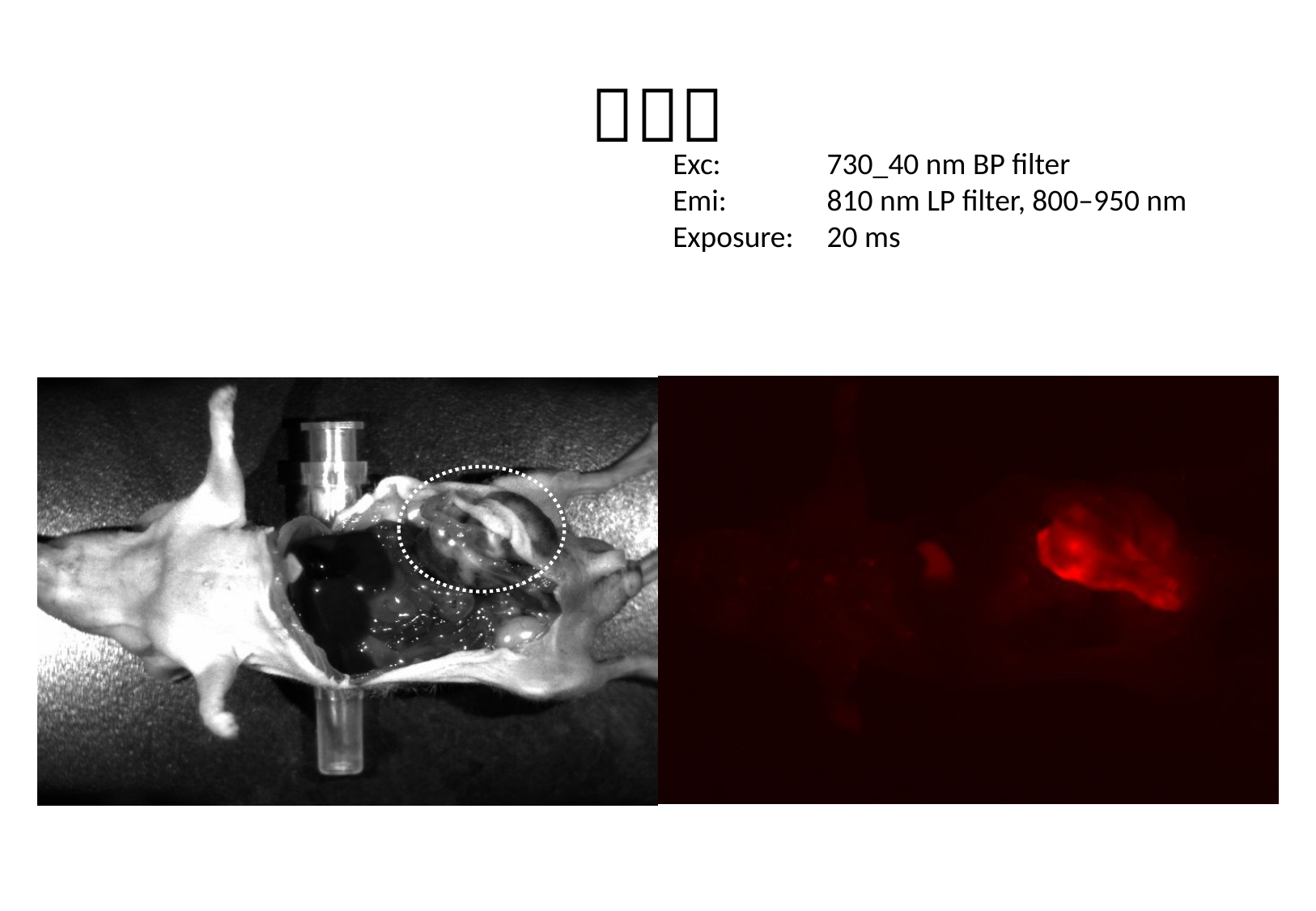

# 開腹後
Exc: 	730_40 nm BP filter
Emi: 	810 nm LP filter, 800–950 nm
Exposure: 	20 ms

## Slide 14
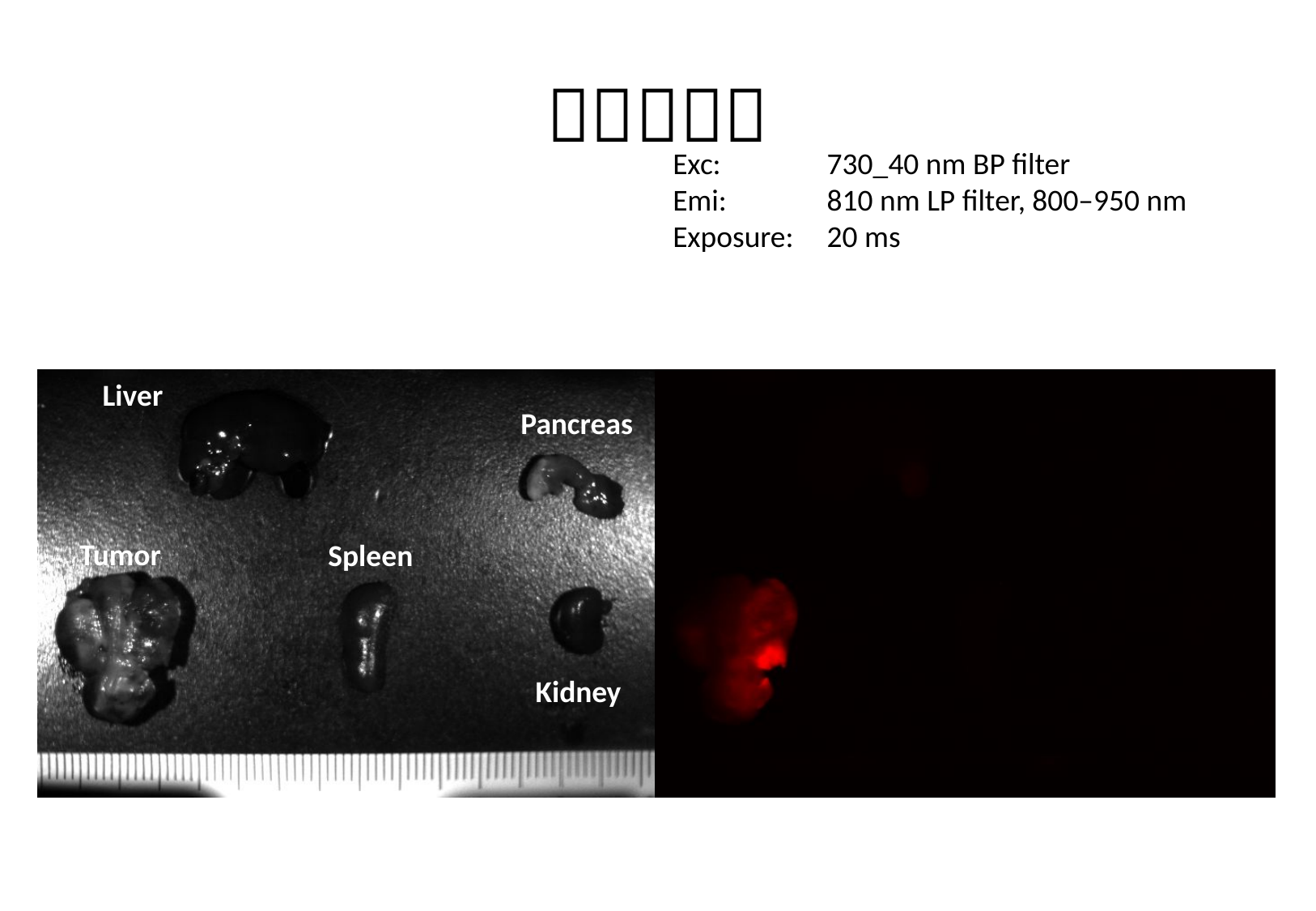

# 臓器摘出後
Exc: 	730_40 nm BP filter
Emi: 	810 nm LP filter, 800–950 nm
Exposure: 	20 ms
Liver
Pancreas
Tumor
Spleen
Kidney
