# Supplementary figures and images for: Photoacoustic Tomography of Human Hepatic Malignancies Using Intraoperative Indocyanine Green Fluorescence Imaging
Source: PLoS One. 2014 Nov 7;9(11):e112667. doi: 10.1371/journal.pone.0112667 (PMC4224503; doi:10.1371/journal.pone.0112667)

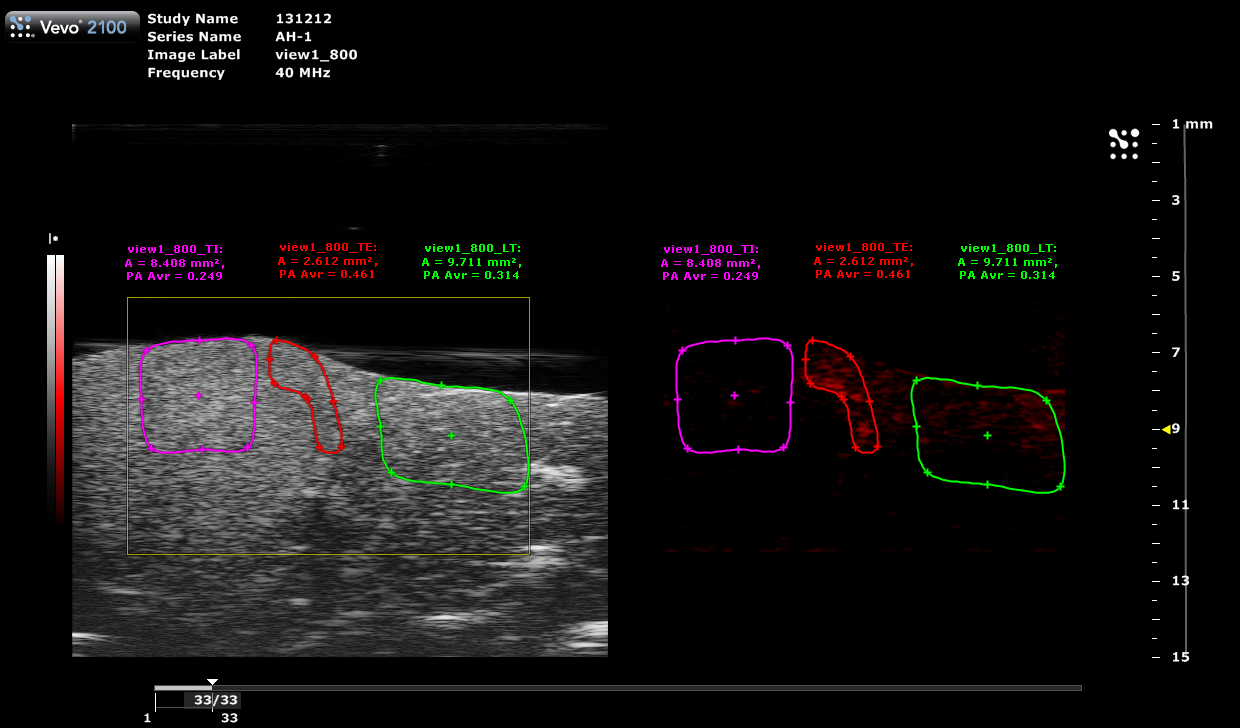

Supplement: Data S1 — Raw data of the present study. (ZIP) [file pone.0112667.s003.zip › SupplementaryMaterials_Ishizawa/AH-1_view1_800_2013-12-12-13-34-36.tif]

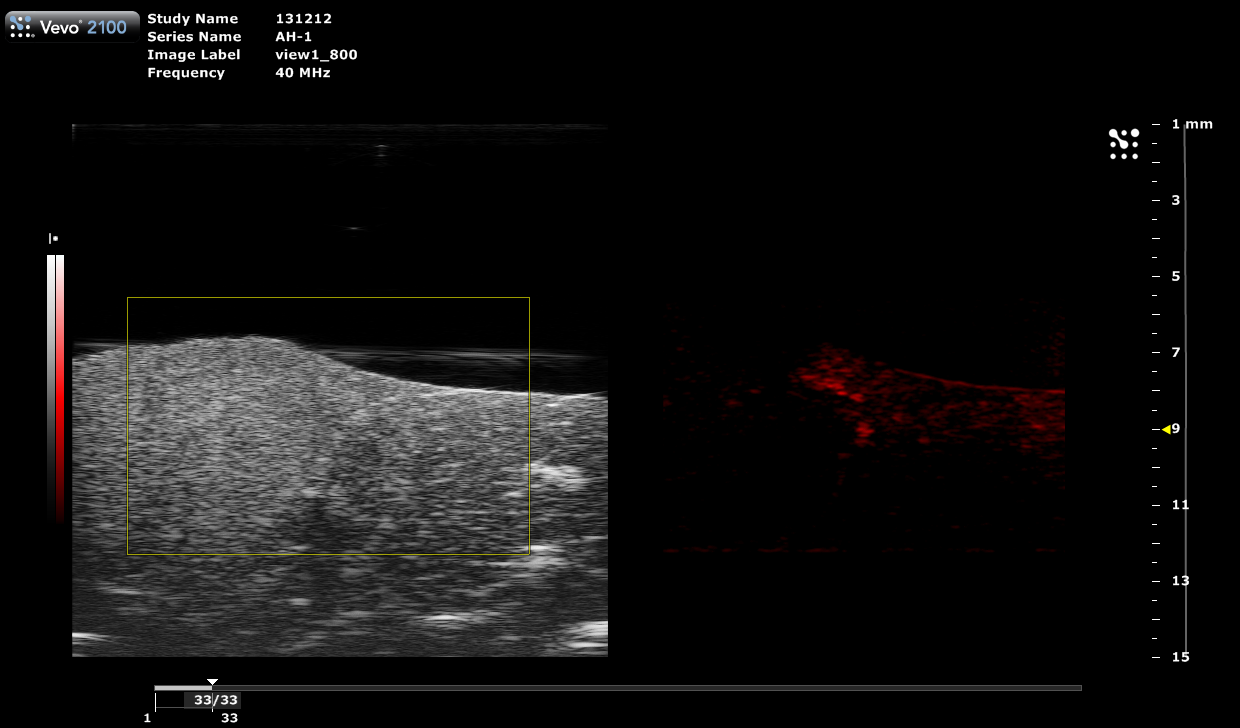

Supplement: Data S1 — Raw data of the present study. (ZIP) [file pone.0112667.s003.zip › SupplementaryMaterials_Ishizawa/AH-1_view1_800_2013-12-12-13-34-36_.tif]

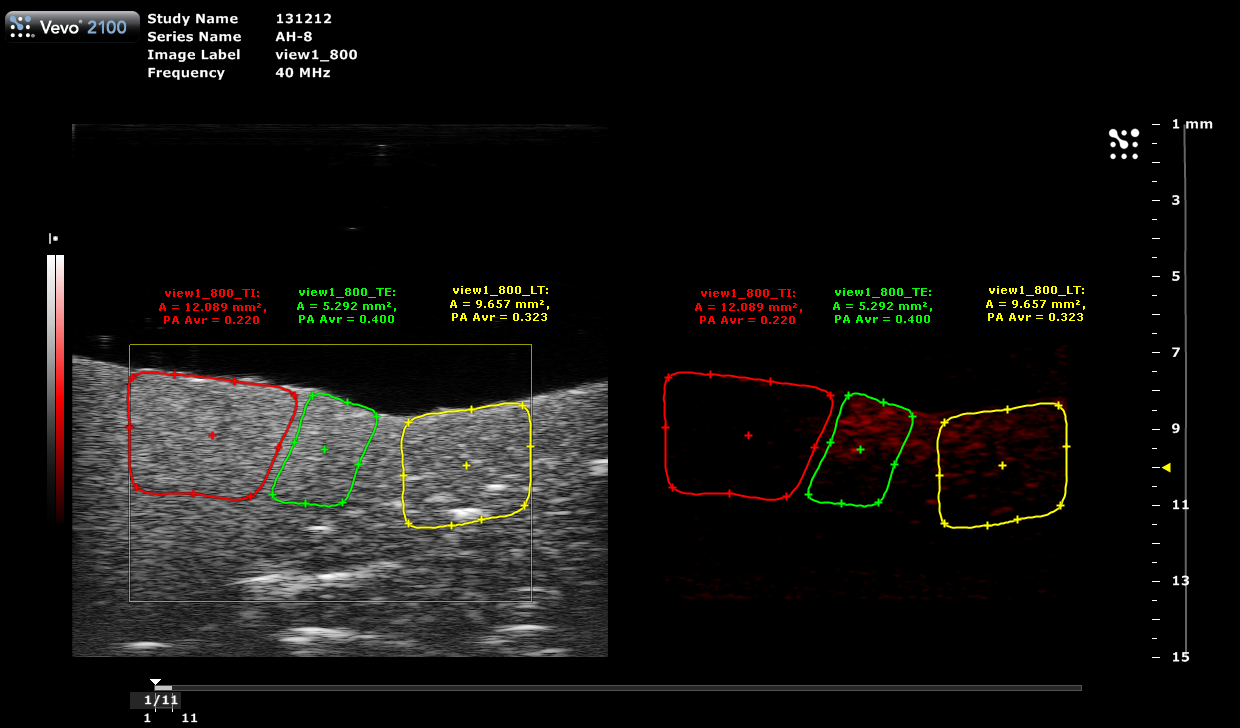

Supplement: Data S1 — Raw data of the present study. (ZIP) [file pone.0112667.s003.zip › SupplementaryMaterials_Ishizawa/AH-8_view1_800_2013-12-12-13-42-19.tif]

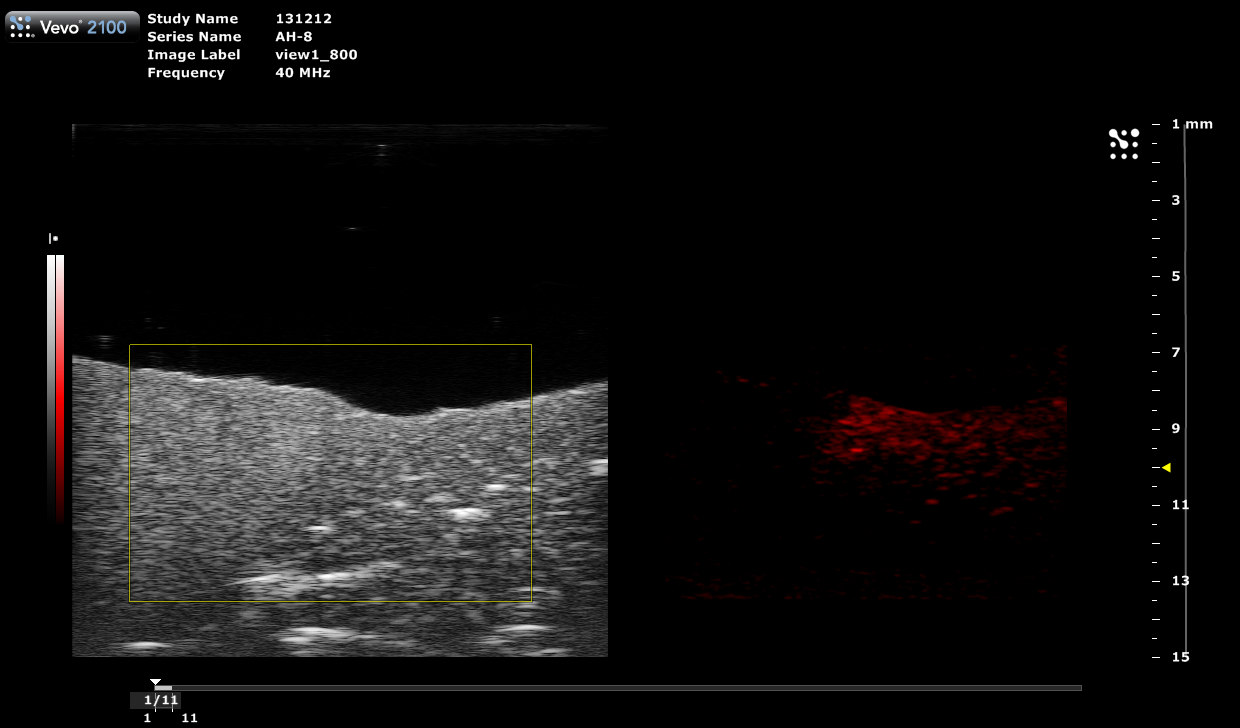

Supplement: Data S1 — Raw data of the present study. (ZIP) [file pone.0112667.s003.zip › SupplementaryMaterials_Ishizawa/AH-8_view1_800_2013-12-12-13-42-19_.tif]

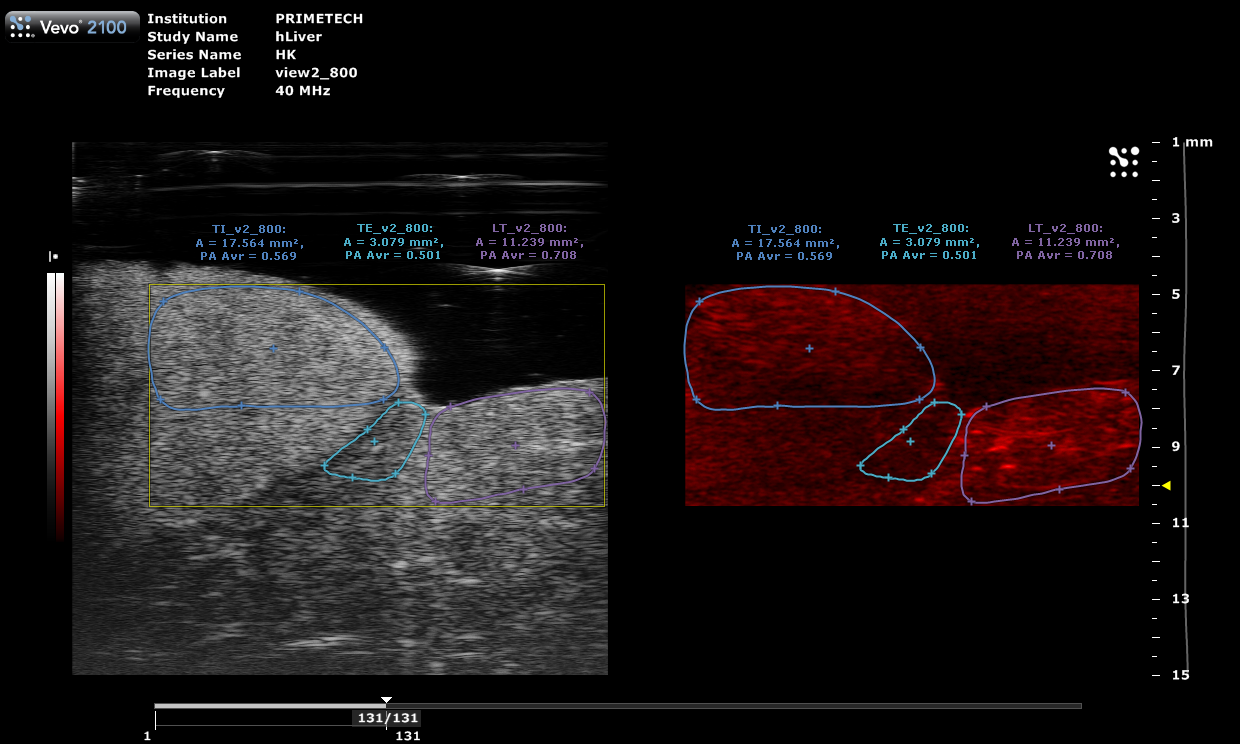

Supplement: Data S1 — Raw data of the present study. (ZIP) [file pone.0112667.s003.zip › SupplementaryMaterials_Ishizawa/HK_view2_800_2013-10-04-16-26-24.tif]

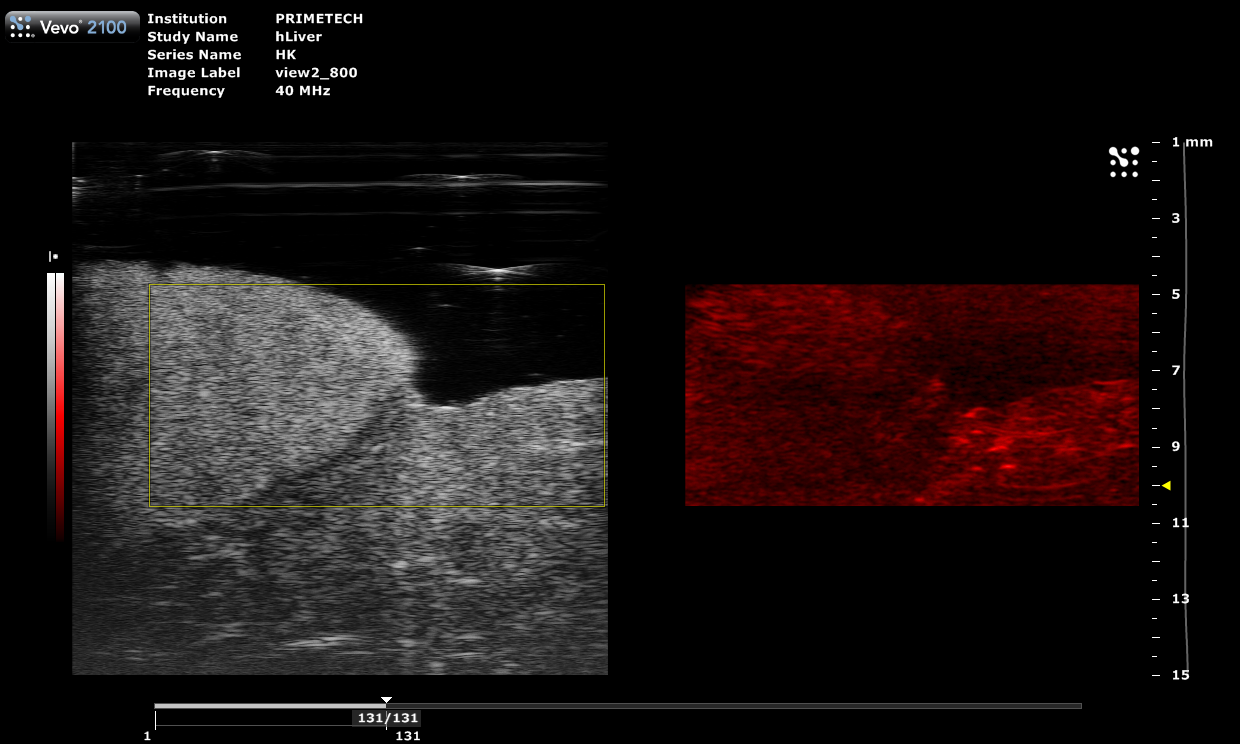

Supplement: Data S1 — Raw data of the present study. (ZIP) [file pone.0112667.s003.zip › SupplementaryMaterials_Ishizawa/HK_view2_800_2013-10-04-16-26-24_.tif]

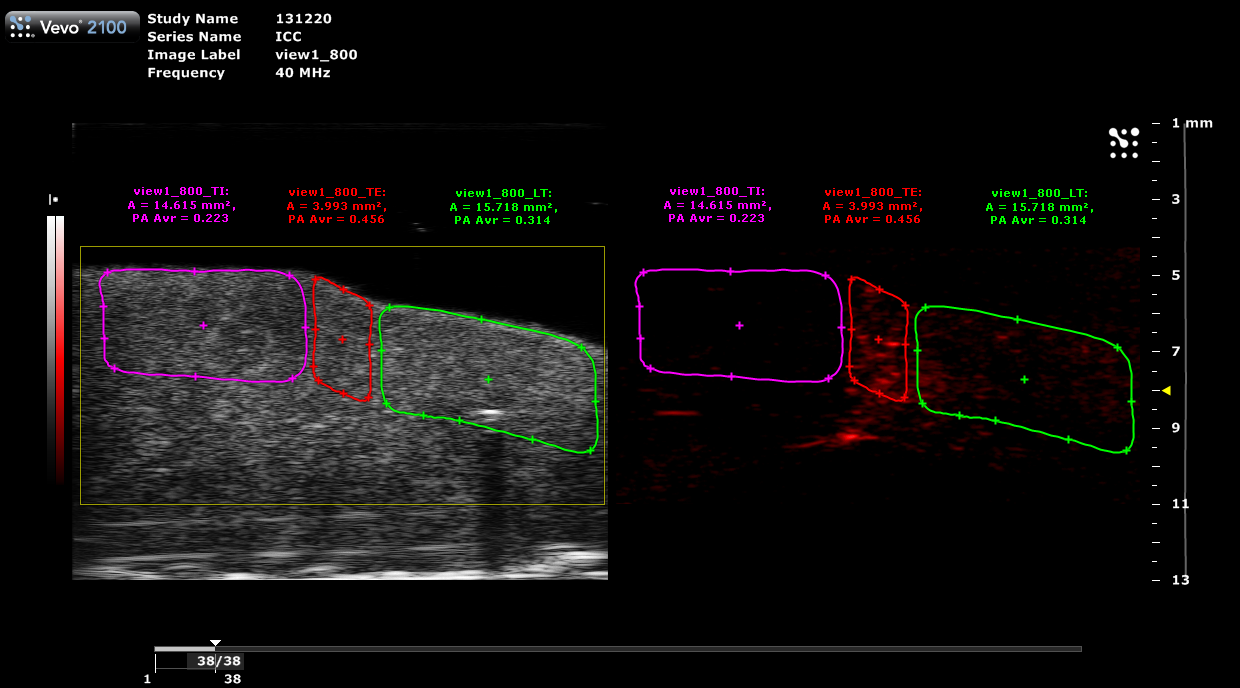

Supplement: Data S1 — Raw data of the present study. (ZIP) [file pone.0112667.s003.zip › SupplementaryMaterials_Ishizawa/ICC_view1_800_2013-12-20-16-41-36.tif]

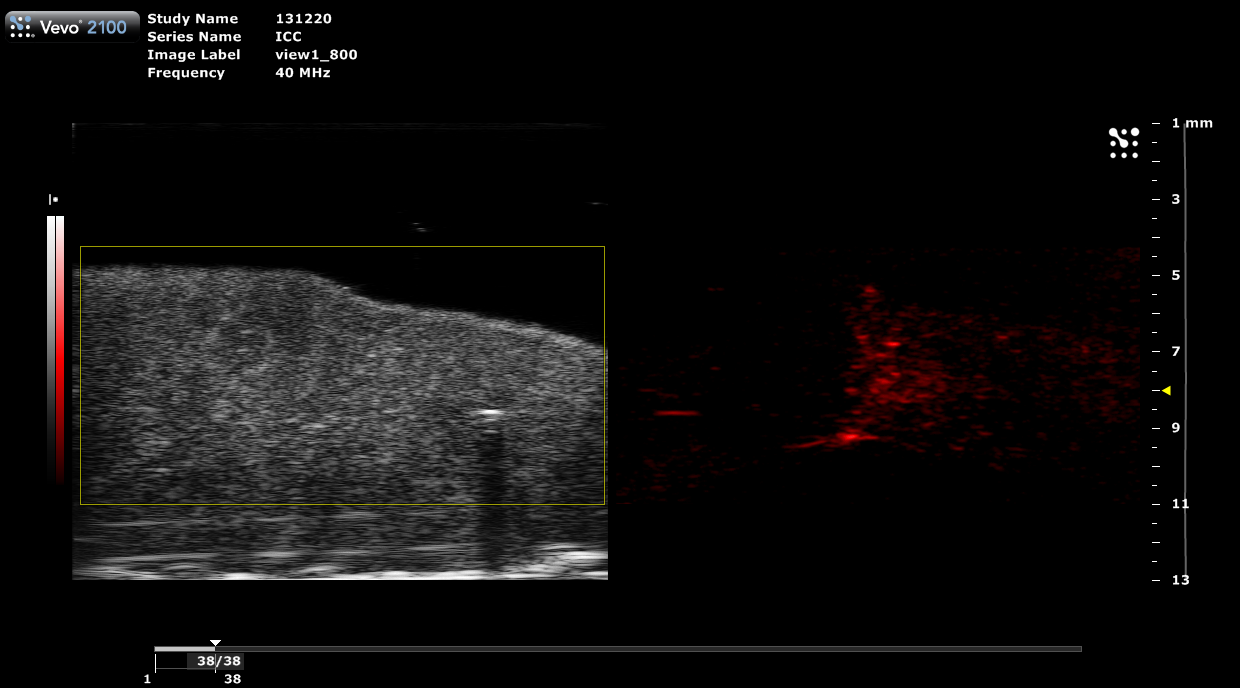

Supplement: Data S1 — Raw data of the present study. (ZIP) [file pone.0112667.s003.zip › SupplementaryMaterials_Ishizawa/ICC_view1_800_2013-12-20-16-41-36_.tif]

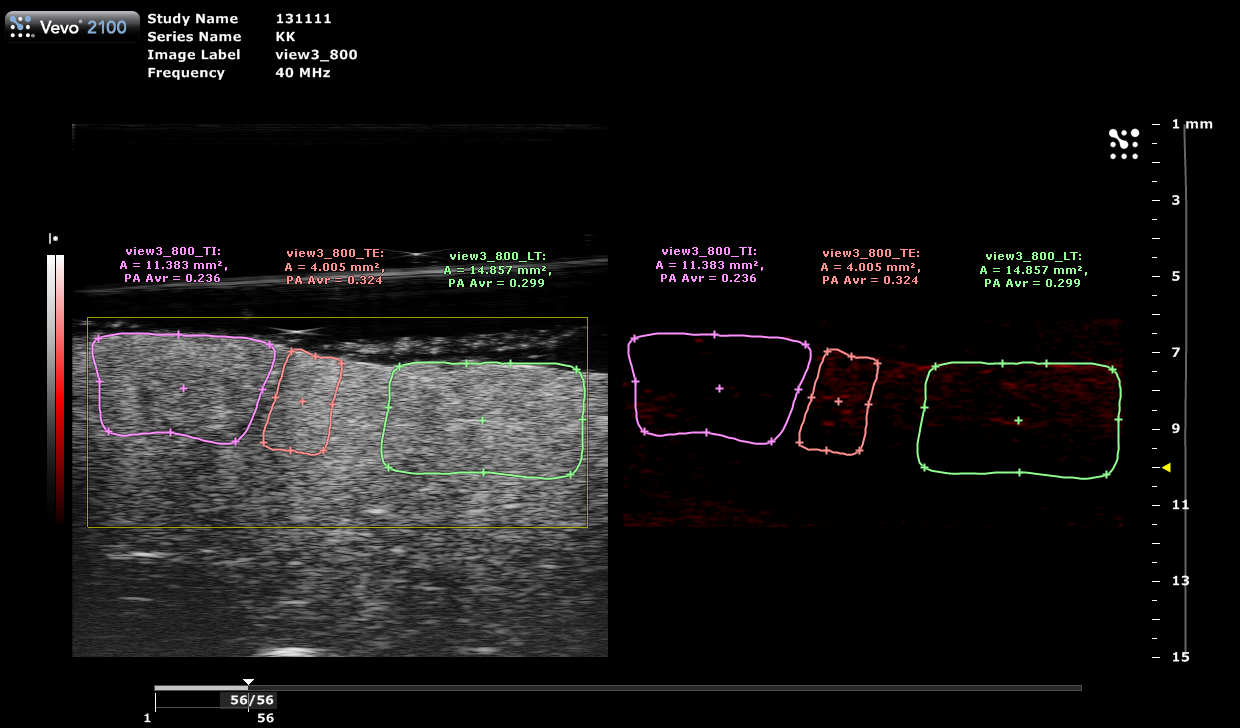

Supplement: Data S1 — Raw data of the present study. (ZIP) [file pone.0112667.s003.zip › SupplementaryMaterials_Ishizawa/KK_view3_800_2013-11-11-16-31-45.tif]

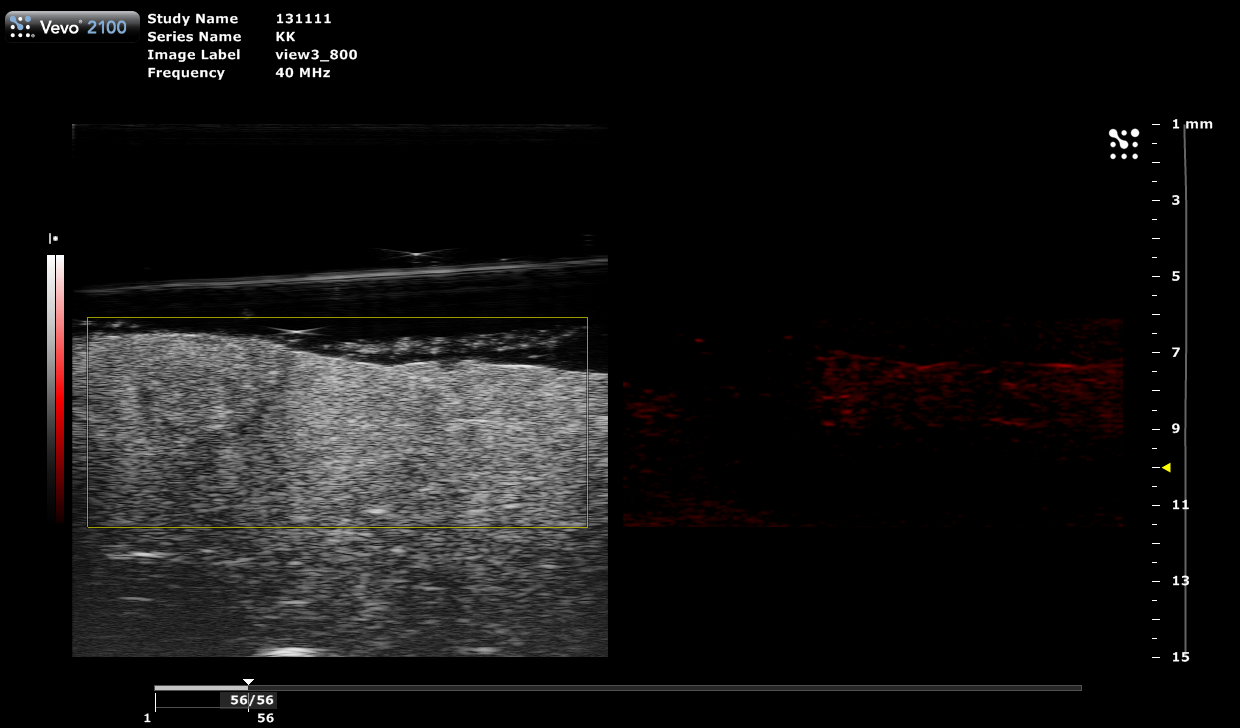

Supplement: Data S1 — Raw data of the present study. (ZIP) [file pone.0112667.s003.zip › SupplementaryMaterials_Ishizawa/KK_view3_800_2013-11-11-16-31-45_.tif]

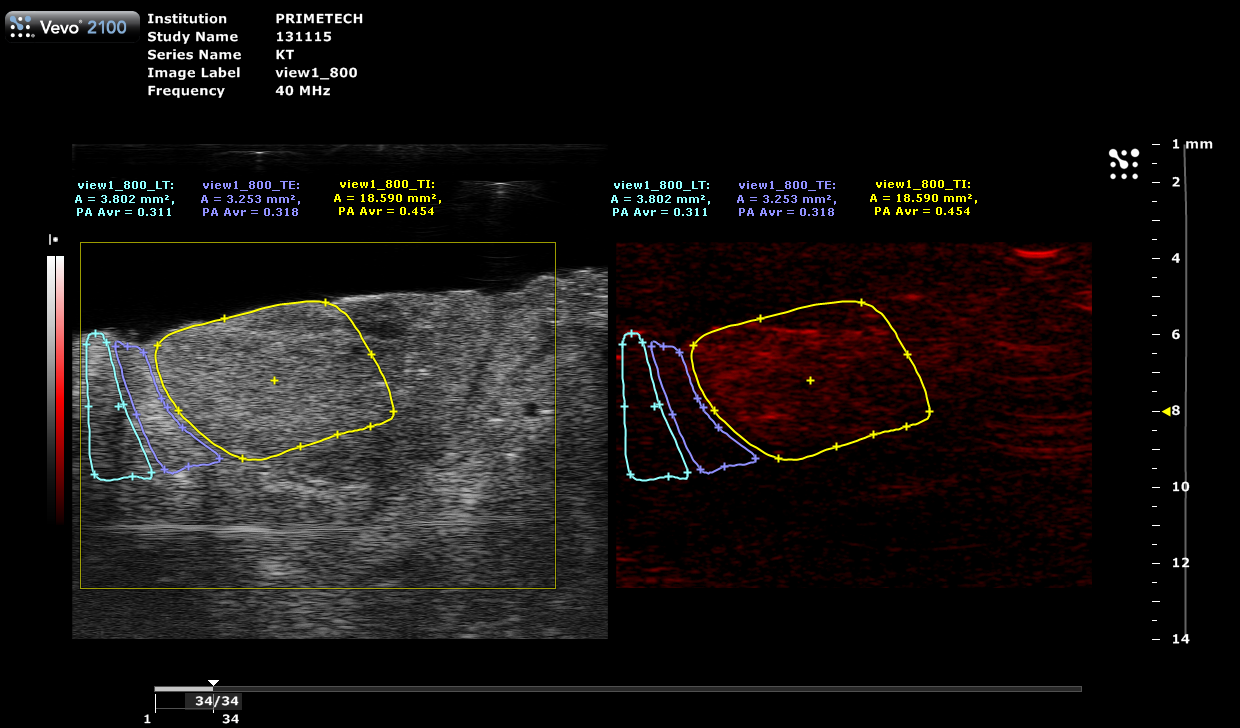

Supplement: Data S1 — Raw data of the present study. (ZIP) [file pone.0112667.s003.zip › SupplementaryMaterials_Ishizawa/KT_view1_800_2013-11-15-14-37-26.tif]

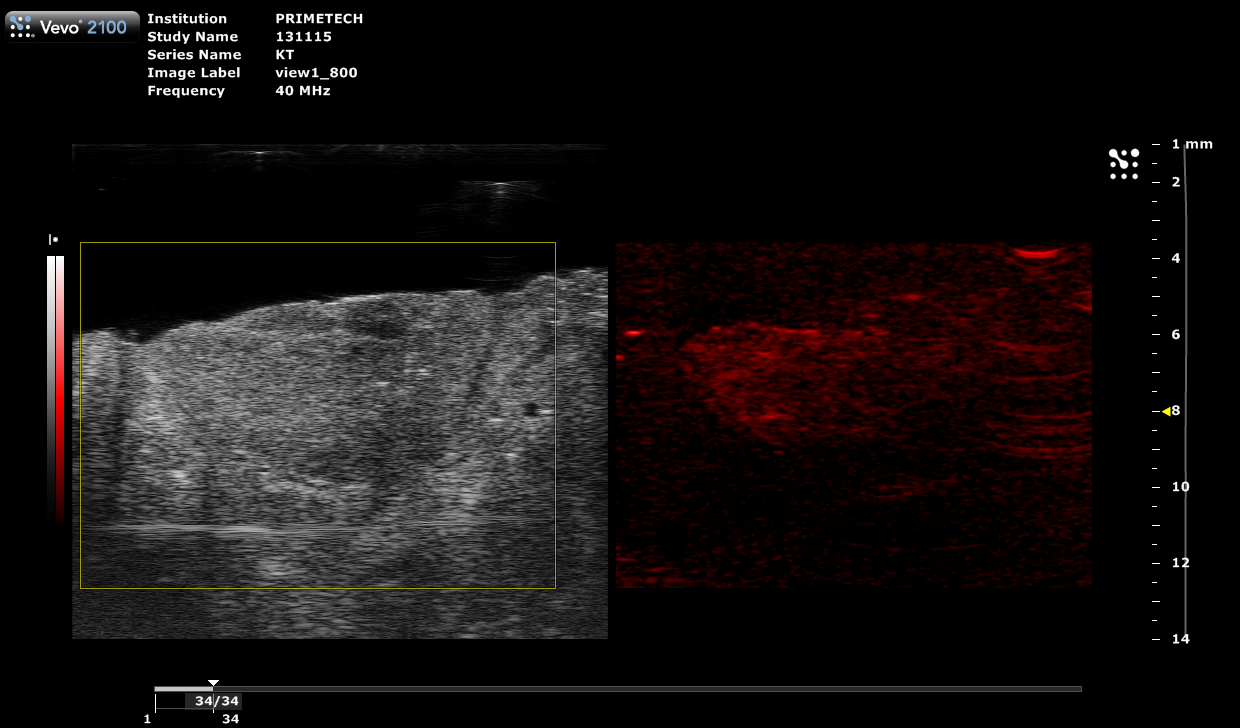

Supplement: Data S1 — Raw data of the present study. (ZIP) [file pone.0112667.s003.zip › SupplementaryMaterials_Ishizawa/KT_view1_800_2013-11-15-14-37-26_.tif]

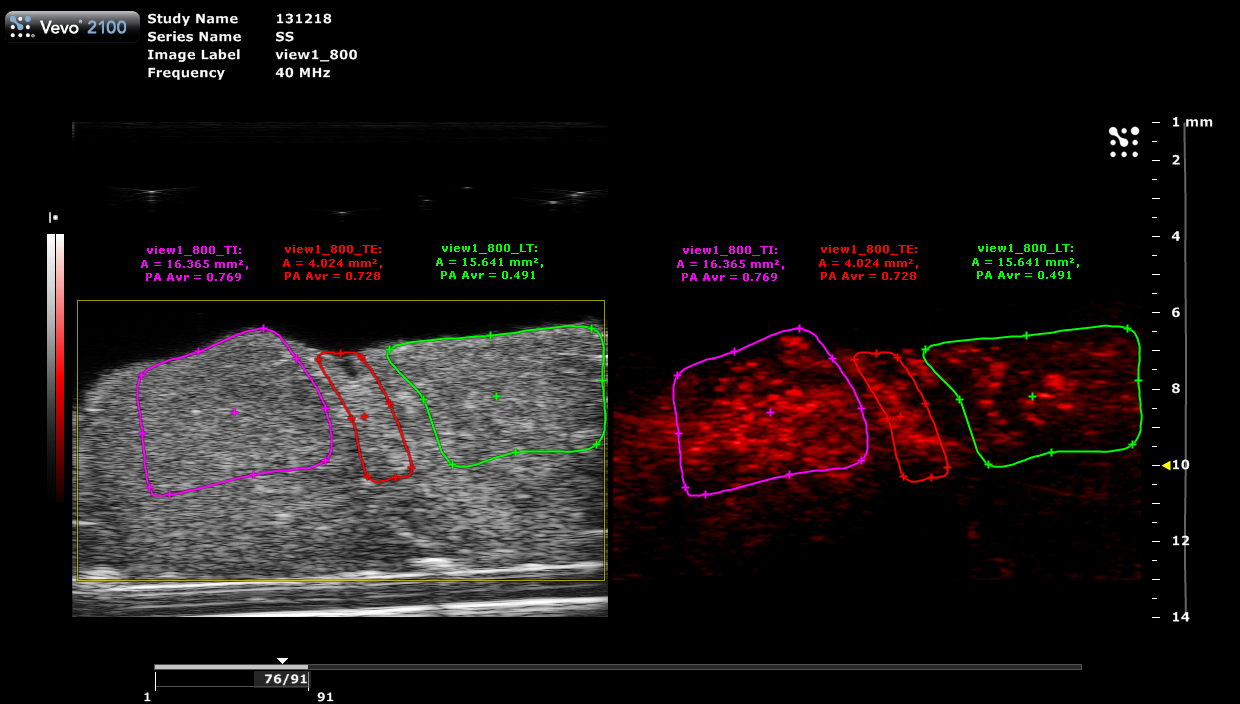

Supplement: Data S1 — Raw data of the present study. (ZIP) [file pone.0112667.s003.zip › SupplementaryMaterials_Ishizawa/SS_view1_800_2013-12-18-16-39-13.tif]

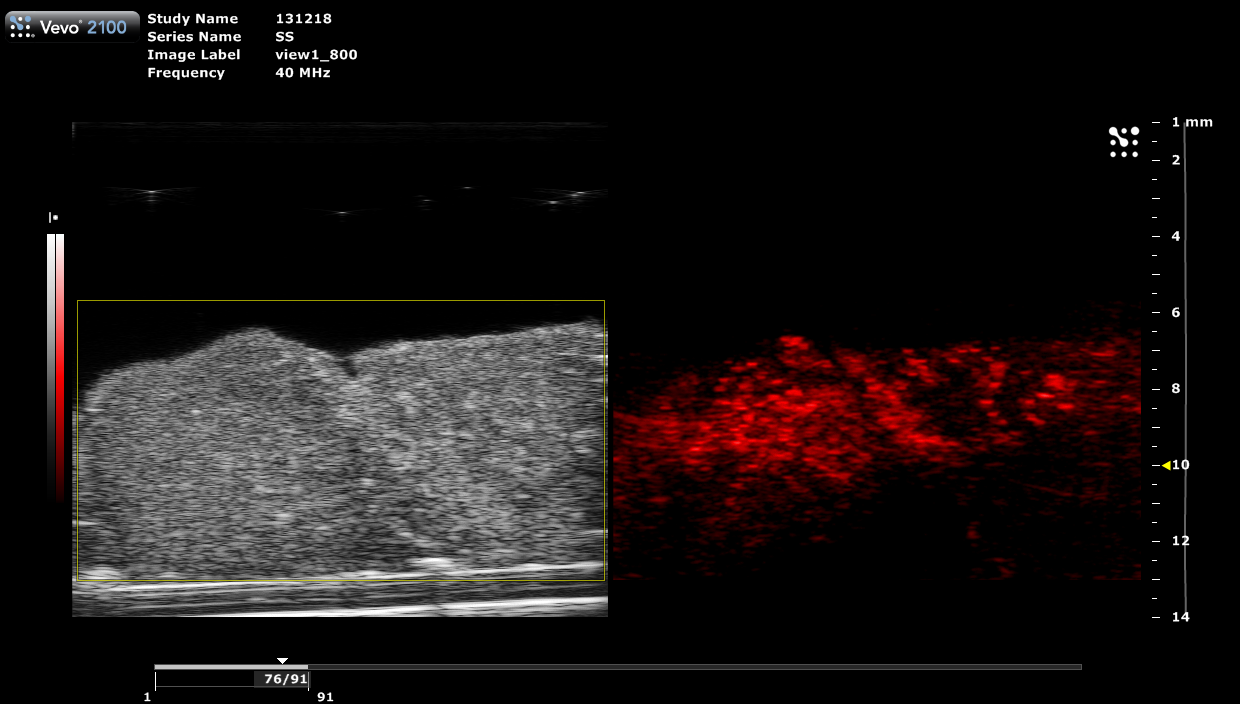

Supplement: Data S1 — Raw data of the present study. (ZIP) [file pone.0112667.s003.zip › SupplementaryMaterials_Ishizawa/SS_view1_800_2013-12-18-16-39-13_.tif]

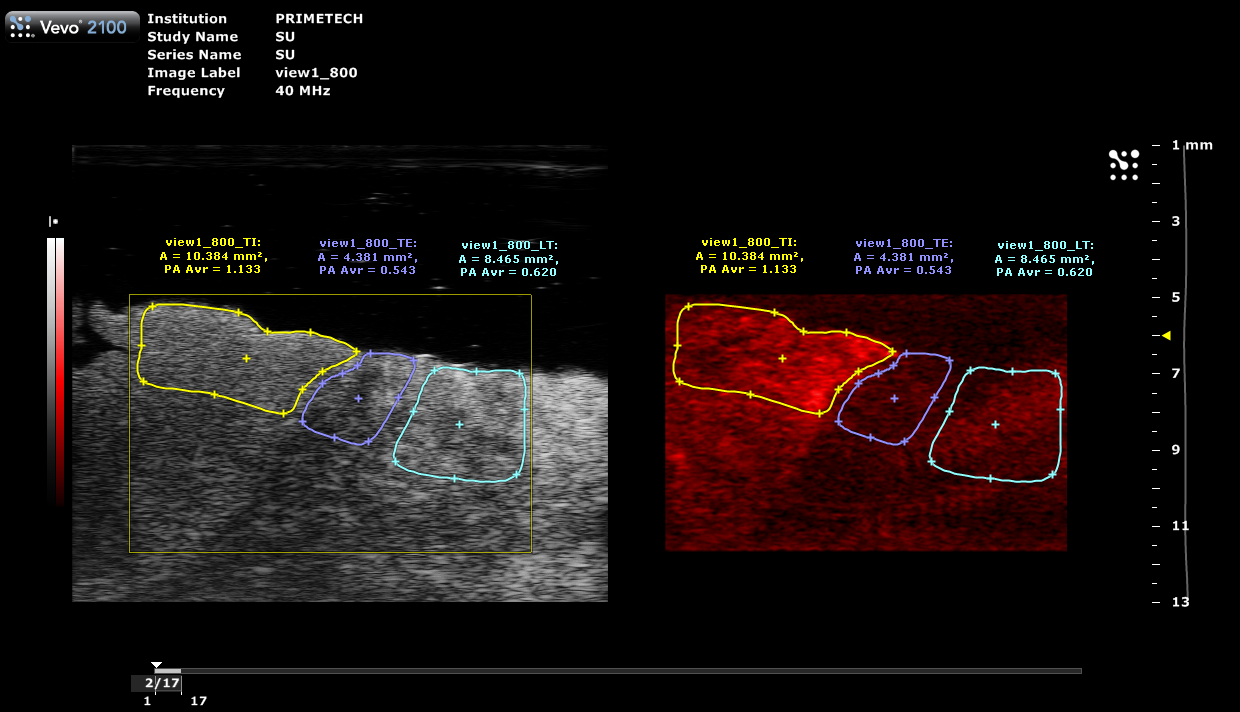

Supplement: Data S1 — Raw data of the present study. (ZIP) [file pone.0112667.s003.zip › SupplementaryMaterials_Ishizawa/SU_view1_800_2013-10-09-14-01-07.tif]

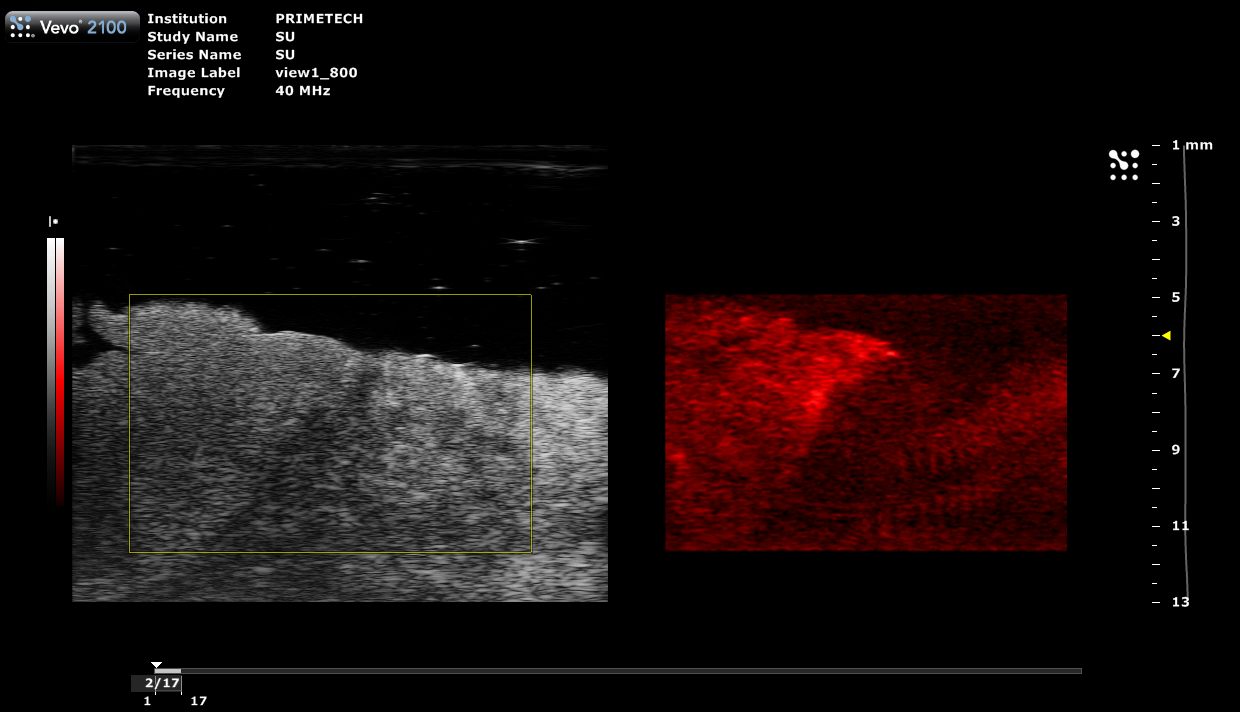

Supplement: Data S1 — Raw data of the present study. (ZIP) [file pone.0112667.s003.zip › SupplementaryMaterials_Ishizawa/SU_view1_800_2013-10-09-14-01-07_.tif]

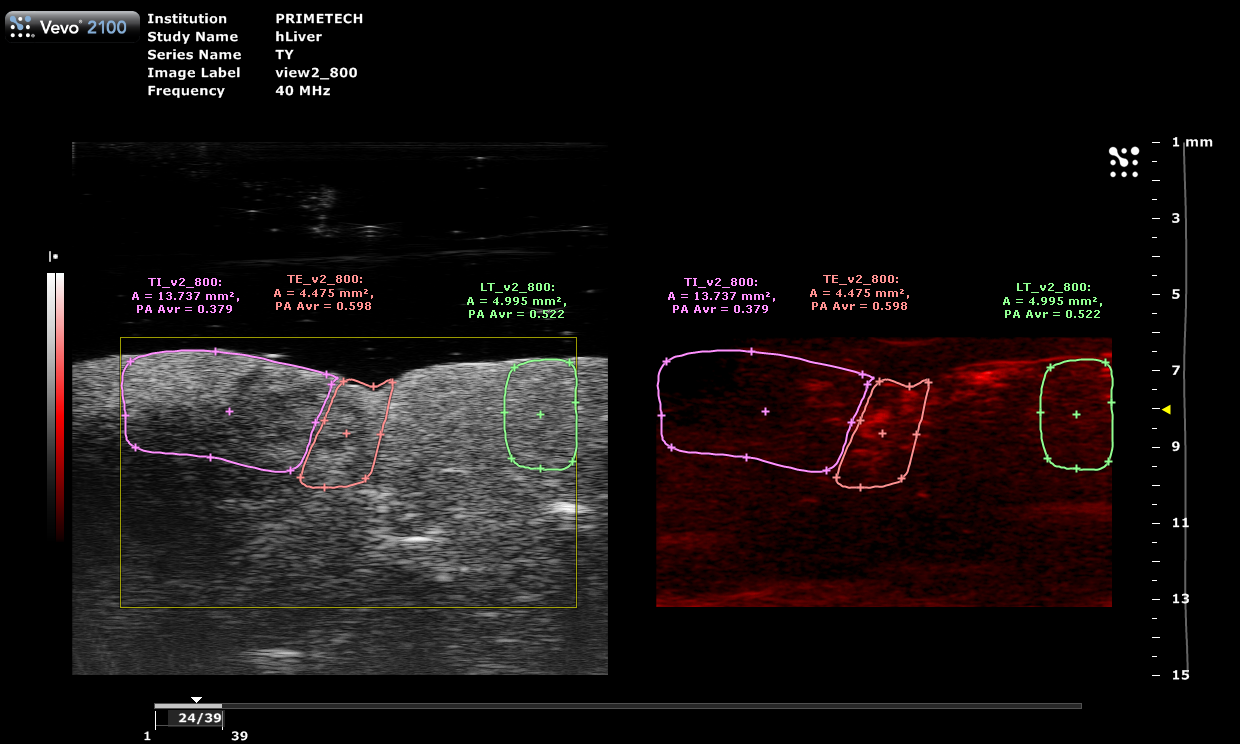

Supplement: Data S1 — Raw data of the present study. (ZIP) [file pone.0112667.s003.zip › SupplementaryMaterials_Ishizawa/TY_view2_800_2013-10-04-17-25-13.tif]

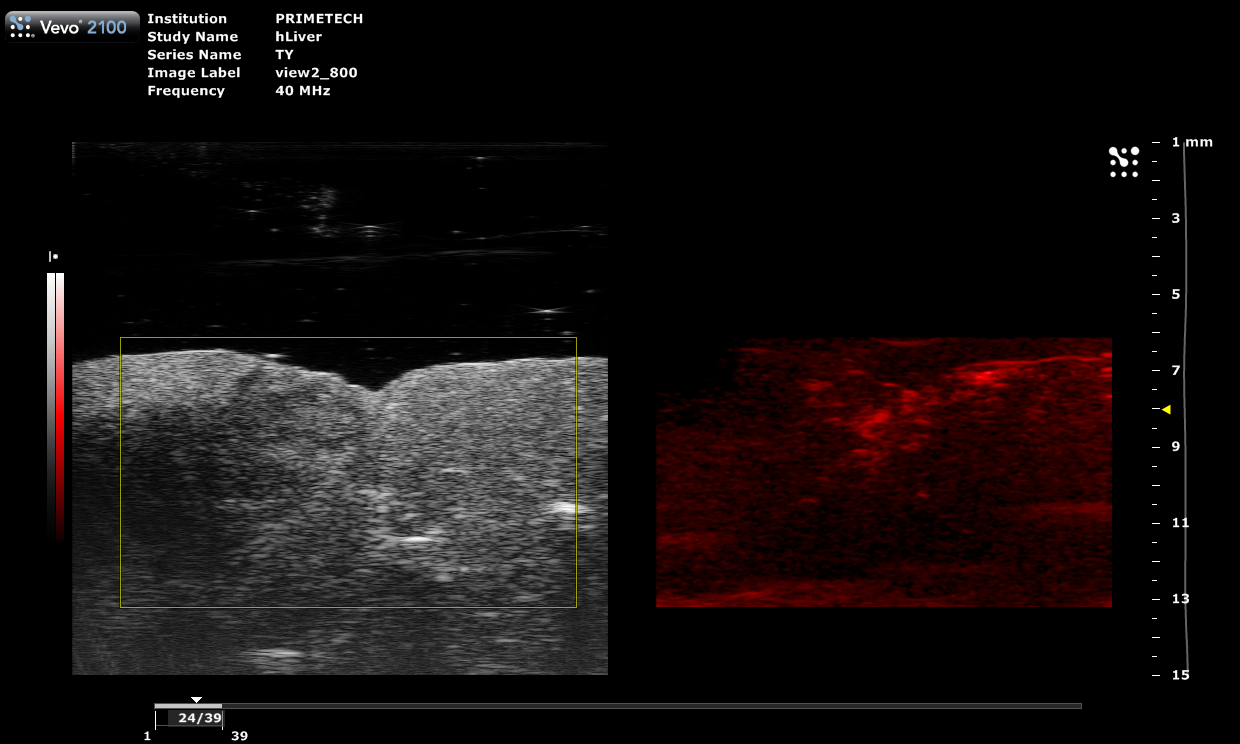

Supplement: Data S1 — Raw data of the present study. (ZIP) [file pone.0112667.s003.zip › SupplementaryMaterials_Ishizawa/TY_view2_800_2013-10-04-17-25-13_.tif]

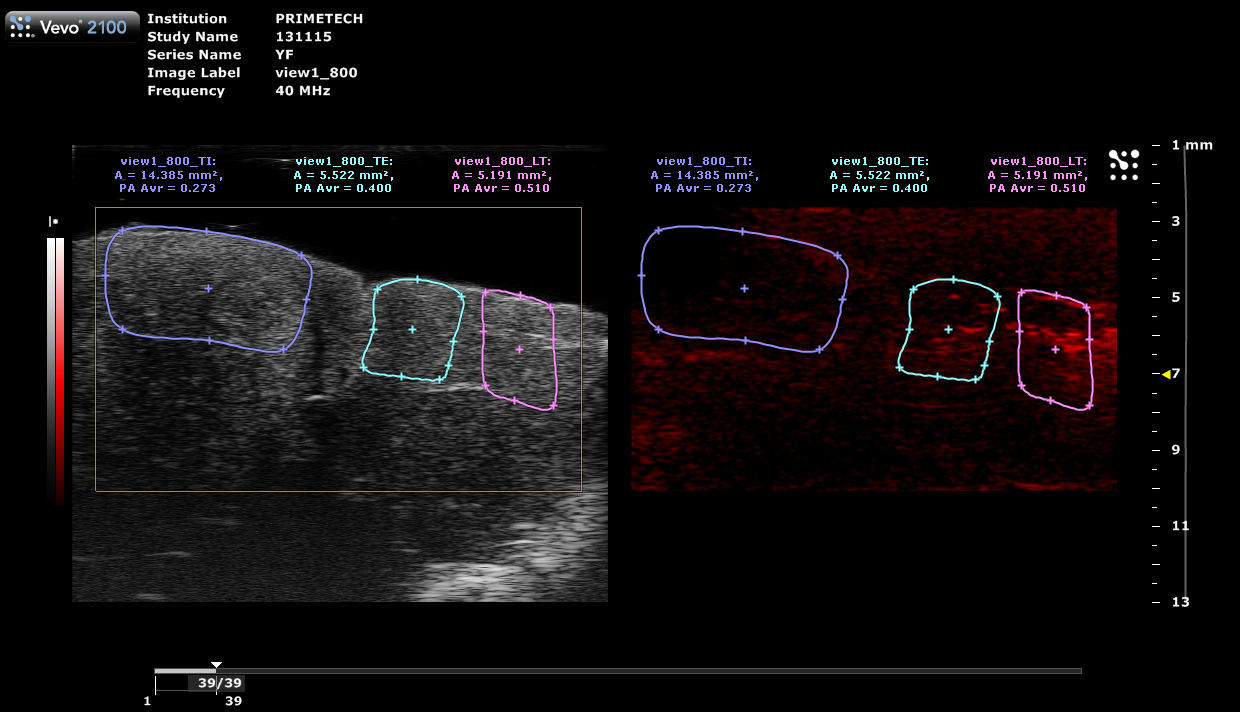

Supplement: Data S1 — Raw data of the present study. (ZIP) [file pone.0112667.s003.zip › SupplementaryMaterials_Ishizawa/YF_view1_800_2013-11-15-15-00-22.tif]

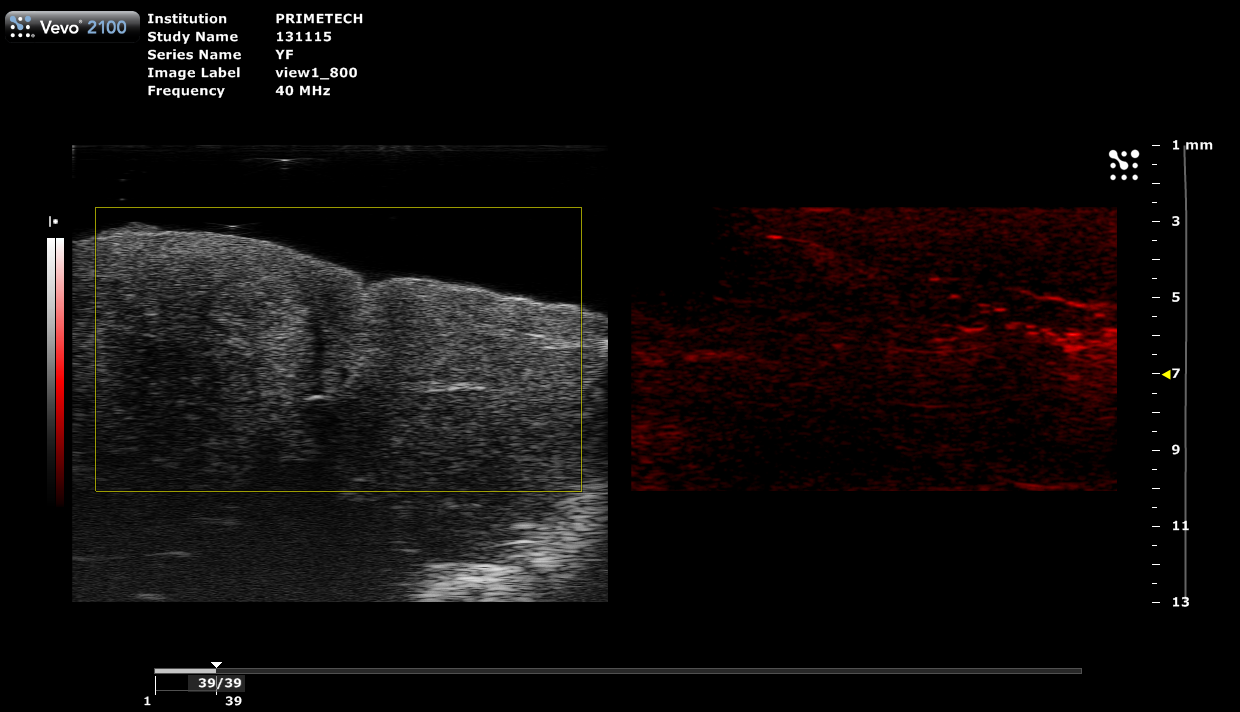

Supplement: Data S1 — Raw data of the present study. (ZIP) [file pone.0112667.s003.zip › SupplementaryMaterials_Ishizawa/YF_view1_800_2013-11-15-15-00-22_.tif]
